# Methodology for a Comprehensive Health Impact Assessment in Water Supply and Sanitation Programmes for Brazil

**DOI:** 10.3390/ijerph191912776

**Published:** 2022-10-06

**Authors:** Débora Cynamon Kligerman, Telma Abdalla de Oliveira Cardoso, Simone Cynamon Cohen, Déborah Chein Bueno de Azevedo, Graziella de Araújo Toledo, Ana Paula Chein Bueno de Azevedo, Susanne M. Charlesworth

**Affiliations:** 1 Departamento de Saneamento e Saúde Ambiental (DSSA)(ENSP), Fundação Oswaldo Cruz, FIOCRUZ, Rio de Janeiro 21041-210, Brazil; 2 Núcleo de Biossegurança (NUBIO) (ENSP), Fundação Oswaldo Cruz, FIOCRUZ, Rio de Janeiro 21040-361, Brazil; 3 Núcleo de Tecnologia e Logística em Saúde (NUTEC) (ENSP), Fundação Oswaldo Cruz, FIOCRUZ, Rio de Janeiro 21040-361, Brazil; 4 Centre for Agroecology, Water and Resilience, Coventry University, Coventry CV8 3LG, UK

**Keywords:** health impact assessment, social determinants of health, sanitation, evaluation methodology

## Abstract

Based on the broader concept of health proposed by the Pan-American Health Organization/World Health Organization (PAHO/ WHO), 2018, and the absence in the literature of indices that translate the causal relationship between sanitation and health, a methodology for assessing the health impact of a water and sanitation programmes, known as a Health Impact Assessment (HIA), was developed, specifically in the Brazilian context, and focused on a school in the northeast of the country. Through exploratory and descriptive evidence, and using documentary research as a method, a retrospective survey was carried out from 2000 to 2022 using documents proposing evaluation methodologies. A single document was found to fit the research objective, which was used to develop the proposed HIA methodology. Development of the methodology consisted of two stages: definition of the health dimensions and selection of the indicators making up each dimension. The HIA methodology was then applied to a school in northeast Brazil to test its use, before a water-efficient management intervention was going to be used. The overall score of 46% indicated that there was room for improvement, which the new management approach could facilitate. This methodology is therefore proposed to be an instrument for the evaluation of public water and sanitation policies, thus assisting managers in the decision-making process and in guiding sanitation programs and plans.

## 1. Introduction

The health-sanitation approach is related to the level of social development of a country [1]. Where there is inadequate water supply and sanitation services, it is likely that indicators of health are poor, reflecting the country’s low economic development. This highlights the importance of establishing the impact of water supply and sanitation programmes on health [1]. However, how is this relationship to be established if there is no unified concept of health? While sanitation is basically established as access to facilities and services that provide collection, transport, treatment, and disposal of human excreta, wastewater, and solid waste [2], definitions of health range from the limited concept of “absence of disease” to the more comprehensive concept of “state of complete physical, mental and social well-being” [3]. However, [4] states that the World Health Organisation (WHO) definition of health is not fit for purpose, and instead proposes that it is defined as “…the experience of physical and psychological well-being.” Additionally, it states that “Good health and poor health do not occur as a dichotomy, but as a continuum”.

**Figure 1:**
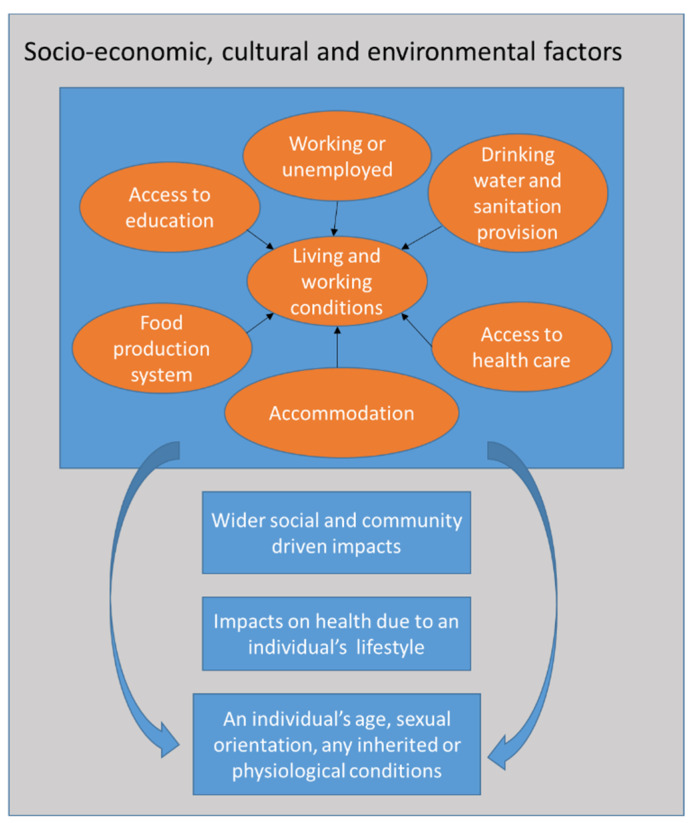
Socioeconomic, cultural, and environmental factors impacting an individual’s health. After Dahlgren and Whitehead, 1991 in [8].

Health is therefore a complex, multidimensional, and dynamic concept, described by [5] as “… resulting from the conditions of food, education, income, environment, work, transportation, employment, leisure, freedom, access to and possession of land, and access to health services…” At the level of the individual, the status of their health can be established by identifying those aspects which need to be considered as a whole [6] and can be considered as determinants [7]. It is therefore quite a wide concept, and thus, this paper proposes a structure to characterise health based on identifying its various dimensions. According to the UK Government [8], and based on the Dahlgren and Whitehead, 1991 model (Figure 1), these factors can be divided into three groups: social, economic, and environmental. Among the environmental determinants, sanitation is one of the most important. 

Provision of adequate water, sanitation, and health is related to the concept of quality of life, which is considered to be “an eminently human notion” related to the satisfaction found in family, love, and social and environmental life, synthesising of all the elements that a society considers its standards of comfort and well-being [9]. It is acknowledged, however, that there are many different location-specific definitions of quality of life from an individual and societal point of view, which are beyond the scope of this paper. Provision of adequate water and sanitation impacts quality of life, as it contributes to positive impacts on the health and well-being of the benefited population [10]. The difficulty of disadvantaged populations accessing adequate water and sanitation infrastructure makes them vulnerable, particularly when these aspects are taken in combination, leading to a low quality of life, emphasising social inequality and allowing the proliferation of diseases related to inadequate sanitation [10]. These diseases include those associated with diarrhoea such as cholera and dysentery, as well as typhoid, intestinal worm infections and polio. The WHO [11] also states that inadequate sanitation can lead to resistance to antimicrobial treatments and can exacerbate stunted growth. 

In general, “sanitation” is defined as being able to access the means to safely dispose of human waste (blackwater containing faeces and urine), which usually means provision of toilet facilities to also include the disposal of menstrual blood, the collection and disposal of solid waste, the management of industrial/hazardous waste, and the treatment and disposal of wastewater [12,13]. The latter includes any water produced by households which does not go into the toilet, such as water from personal washing, clothes washing, kitchen preparation, etc., and is also commonly called “greywater”. Thus, assessing the impact on health of improvements in sanitation conditions is important because it measures the effectiveness of this type of action on the quality of life of the benefited populations, even assisting with monitoring the morbidity and mortality rates of related diseases. This impact can be measured in several ways, including by the population served, as carried out by the Brazilian Ministry of Regional Development, or by the length of the network implemented [14], although this does not account for small scale, decentralized systems, or the quality of the service provided. The Ministry of Health measures the incidence of water-borne disease, and thus there is little interaction between these organisations when it comes to measuring the impact on health of sanitation actions. 

The Demographic and Health Surveys (DHS) and Multiple Indicator Cluster Surveys (MICS) are worldwide sources of information on the health status of children and women carried out by the United Nations Children’s Fund (UNICEF). DHS covers selected nutritional indicators, fertility, and issues of health around reproduction, maternal and child health, HIV and AIDS, maternal and child mortality, malaria, and other indicators. MICS covers health, education status, protection of children, and prevalence of HIV/AIDS according to geographic, social and demographic characteristics (https://inddex.nutrition.tufts.edu/data4diets/data-source/demographic-and-health-surveys-dhs-multiple-indicator-cluster-surveys-mics, accessed on 30 September 2022). These data are statistically robust and comparable globally [15]; however, a survey of the online databases did not include Brazil and did not include the subject of sanitation. The MICS surveys were carried out at intervals, and in the case of Brazil, there are records for 1986 (MICS1) [16], 1991 (MICS2) [17] and 1996 (MICS3) [18]. There should have been a further survey, or MICS4, between 2010 and 2012, but Brazil was one of the countries, including India, which did not carry this out [15], which may explain their absence from the UNICEF databases. The information contained in the MICS1-3 databases are specific to Brazil and are therefore in Portuguese. Sanitation was not considered during the first two MICS; however, by MICS3, sanitation was considered as it was reported that for the country overall, whilst over 70% of households had access to a water supply, just over 40% of them did not have a bathroom and were not connected to a sewerage network. Table 1 shows the breakdown of this data by region and land use, and illustrates that whilst urban areas do have nearly 85% coverage for water supply, nonetheless, over half were not connected to a sewerage network, and may not have had a bathroom. In comparison with the urban areas, Table 1 shows that rural households in the 1996 MICS3 had far less access to a water supply (nearly 25%), and only 6.3% had a bathroom or were connected to a sewerage network. In comparison with other areas of Brazil, the north of the country had the lowest % of all households with a bathroom or connection to a sewerage management system; this may reflect the population density, which is very small. There are areas where there is just one inhabitant per hectare, and as a consequence, it is not possible to install a sewage system; instead, a septic tank is used for each individual household.

The United Nations Millennium Development Goal (MDG) sanitation target was to halve the number of people who did not have access to basic sanitation (water and sanitation) by 2015 [19]; however, it is debatable whether this was achieved [19,20]. Superseded by the United Nations’ Sustainable Development Goals (SDG) [21], the current ambition is that everyone will have “adequate and equitable” water and sanitation and basic hygiene by 2030. However, as stated by [12], “the world is alarmingly off track to deliver sanitation for all by 2030” (p5). However, authors such as Pereira and Marques have recently asked the question of SDG6 (the “water” SDG) “Are we there yet?” [22] deciding that UN Member States were in fact closer than widely perceived, in that the gap between the Best and Worst Performance Frontiers had closed, although in [23] they did acknowledge that in the specific case of Brazil, the gap had widened.

Additionally, because the targets set by the SDGs are global and aspirational, in essence, each country is individually responsible for organising specific mechanisms to achieve them, and there are no country-specific metrics to enable the achievement of these goals. According to [24], there are several countries which are now not using best practice in order to achieve the aims of SDG 6; Brazil is second to China for this record in terms of its size and population. As a result, the Brazilian National Sanitation Information System reported that 16% of the population of Brazil cannot access a water supply network, 46% are not connected to a sanitation network, and 22% of the wastewater produced is not treated [24]. According to Cavalcanti et al. [25] by improving the performance of those companies involved in the integrated management of basic sanitation, and thereby addressing these issues, there is potential for access to a sanitation network across Brazil to increase to 76.5%. Ferreira et al. [26] identify that the provision of adequate drinking water facilities would improve human health by reducing the numbers of hospitalisations due to water-related disease, could positively impact the whole population, and would bring Brazil closer to the situation in developed countries.

In Brazil, the National Guidelines for Basic Sanitation, established by Law nº 14026 (2020) [27] which address, among other aspects, issues around drinking water and sanitary sewerage. These services must be provided based on principles such as: universal access; completeness; safety, quality and protection of the environment and public health; availability; local specificities; economic efficiency and sustainability; and communication with other policies aimed at improving quality of life, for which basic sanitation is a determining factor. However, this is a law, and as such it did not present a methodology for measuring the impacts resulting from the implementation of basic sanitation actions. This, therefore, goes some way towards justifying the proposition of the methodology for evaluating the health impact specifically of sanitation programs in the Brazilian context, a comprehensive Health Impact Assessment (HIA). A study by Abe and Miraglia [28] found that the use of the HIA approach was not common across Latin America in general or Brazil in particular, although Thandoo et al., [29] acknowledge that, across Latin America as a whole, only Mexico and Brazil have published HIA guidelines; as these guidelines are published solely in Portuguese, they are not readily accessible internationally. The authors of [28] also found that the monitoring and subsequent analysis of health impacts was not robust, both of these issues implying that encouragement in the application of HIA was needed. Abe and Miraglia also published a study in 2016 [30] utilising HIA in the Brazilian context, but this was in terms of identifying the impacts of air pollution on health and did not consider water-resource management. Silveira and Fenner [31] also found that HIA was not commonly used in Brazil, particularly highlighting the benefits of engaging with multiple stakeholders in HIA as opposed to the standard Brazilian approach of the environmental licensing process.

In general, HIAs evaluate the absence of disease, taking an epidemiological perspective [30], but the proposed, comprehensive methodology uses seven dimensions of health, not just epidemiology. These seven dimensions are: sanitary, environmental, technological, sociocultural, epidemiological, mental well-being and economic; their development is discussed in Section 3. The present study applies this extended methodology to a situation in Brazil, whereby the water supply system needs to be improved. The relationship between water/sanitation and health is a complex one, affecting all aspects of health. According to Heller [32], there is not a comprehensive enough assessment to support any relationship between water supply and sanitation interventions and health indicators.

The first aim of this article is to propose a HIA methodology which can be used to specifically assess the impacts on human health of water supply and sanitation projects in Brazil but has the potential to be applied elsewhere in the world with similar issues regarding sanitation. This will be achieved by proposing indicators which represent the status of human health in the Brazilian context. Whilst HIA is a well-recognised technique to show the results of suitable interventions to address inadequacies [33], there is a dearth of its application to instances of insufficient provision of water and sanitation in communities, and thus, this study provides an extension to its use in a specific context. This paper therefore begins by considering both qualitative and quantitative impacts related to water supply and sanitation programmes translated into indicators that address the various dimensions of health. By carrying out this comprehensive survey of health dimensions, the HIA produced enables the engagement of multiple stakeholders simultaneously, encouraging dialogue in the development of associated policies and guidelines. The second aim is to test this methodology in a school setting where sanitation is inadequate and to present preliminary, baseline results of applying the HIA before sanitation issues have been addressed.

## 2. Materials and Methods

For the construction of the comprehensive HIA evaluation methodology, exploratory and descriptive research was used by means of documentary evidence. It was characterised as exploratory because it sought to understand problems experienced in the local context, i.e., Brazil specifically, and to characterise them. It was also descriptive to the extent that it detailed each dimension of health and its indicators. Primary sources of information were used, which consisted of official Brazilian reference documents that had not been analysed previously. It is acknowledged that there are many full literature surveys either of global HIA approaches (e.g., Harris-Roxas et al.’s [34] state-of-the-art review) or individual countries worldwide (e.g., Dannenberg [35]); therefore, it was thought to be beyond the scope of this study. Therefore, the Brazilian documents alone provided the basis of the health dimensions to be utilised to construct a comprehensive HIA.

In order to identify methodologies for evaluating the health impacts caused by water supply and sanitation programmes in Brazil, the databases accessed were from the Ministry of Health, Ministry of Environment and Ministry of Cities, currently the Ministry of Regional Development, since they are responsible for establishing standards, proposing, monitoring, and implementing policies, guidelines, and actions for basic sanitation. Since these databases contain Brazilian information, much of the corresponding documentation is not available in English, but only in Portuguese. A retrospective survey was conducted for the period 2000 to 2022 around the publication of the MDGs dating from 2000, and the SDGs which were produced between 2014 and 2015. The following keywords were used as locators: “health impact assessment”, “social determinants of health”, “water supply”, “sanitation” and “assessment methodology”. The inclusion of these criteria was determined from documents that proposed methodologies for assessing health impacts caused by the provision of water supply and sanitation. Exclusion criteria were documents that only evaluated public policies related to water supply and sanitation alone, and did not take account of dimensions of health.

The search was only able to identify three possible sources of information based on the criteria and sources of information given above:

1. “Plano Nacional de Saneamento Básico” (National Basic Sanitation Plan) (PLANSAB) [36], coordinated by the Ministry of Regional Development; in Portuguese, with relevant sections translated into English in Supplementary Material S1.
2. “Pesquisa Nacional de Saneamento Básico” (National Survey on Basic Sanitation) (PNSB) [37], a survey applied by the Brazilian Institute of Geography and Statistics (IBGE); in Portuguese, with relevant sections translated into English in Supplementary Material S1.
3. “Avaliação de Impacto na Saúde das Ações de Saneamento” (Health Impact Evaluation of Sanitation Actions) [38], a methodological proposal by the Pan-American Health Organization/World Health Organization (PAHO/WHO) in Brazil, together with the Ministry of Health in 2014; in Portuguese, with relevant sections translated into English in Supplementary Material S1.

It was observed through the search that evaluation of government programs related to the provision of basic sanitation was lacking, with overviews of local sanitation conditions in certain cities and/or regions and their historical evolution predominating. As PLANSAB [36] and PNSB [37] are public policy evaluation instruments for the development of resource investment plans, they did not present a methodology for the evaluation of sanitation actions and their impacts on health and were therefore excluded. Thus, only the methodology developed by PAHO/ WHO [38], which discussed health dimensions and indicators, was applicable to this study.

The comprehensive Health Impact Assessment methodology comprises the following steps:

a) Definition of the dimensions of health that made up the evaluation methodology.

b) Selection of indicators for each dimension, which represented the variables or attributes, enabling their description and measurement, both quantitatively and qualitatively.

The following need to be performed before and after the intervention to calculate its health impact.

c) Use of evaluation tools for assigning a grade for each indicator.

d) Calculation of the value of each dimension as the average value of its indicators.

e) Indication of the weight for each dimension (depending on the type of sanitation intervention).

f) Calculation of the weighted average of the dimensions, which will be the value of the health condition.

g) After the intervention, calculation of the health impact as the difference between the health condition before and after the intervention.

**Figure 2:**
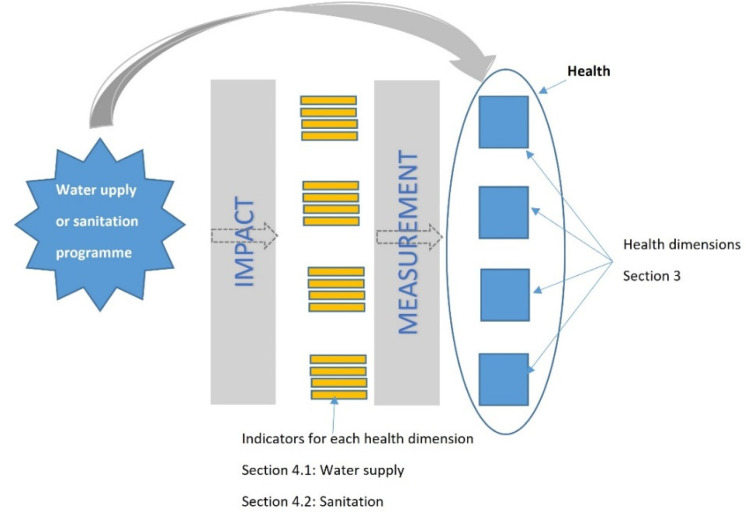
Proposed Methodology for Comprehensive Health Impact Assessment in Water Supply and Sanitation Actions, indicating sections in the paper where information on the dimensions and their indicators can be found.

The HIA methodology tackles the relationship between water supply and sanitation programmes, and health. This methodology uses indicators as a tool to relate the implementation of water supply and sanitation interventions, with improvements in each dimension of health, by assessing how each intervention affected each indicator. The proposed methodology, illustrated in Figure 2, provides a formal and specific measurement of the impact of water supply or sanitation programmes on health improvement. The first step in the methodology was to determine the dimensions of health, followed by determining the indicators that reflect the condition of each dimension. The indicators would depend on the nature and scope of each water supply or sanitation intervention; those related to each health and sanitary dimension are listed in Section 4.1 and Section 4.2. Many indicators can be assessed by comparison with official standards (for example, water-quality determinands for the sanitary dimension), while others can be estimated by expert field analysts, through perception surveys (including epidemiology, mental well-being, the environment, and sociocultural) and information from institutions linked to the health sector both at federal and municipal or local level where the latter has information from family health programs. The technological dimension can be observed on a site visit by examination of the infrastructure, and also in available manuals and guidelines. For the economic dimension, data can be obtained from the municipalities on water consumption, the costs of deliveries by water tanker and the school’s electricity bill, with the health secretariat supplying data on health spend. Further information linked to the environmental dimension includes climate data and geography specifically related to the site [5,38]. Using this methodology, a baseline scenario can be determined, against which it may be possible to assess if there is an associated improvement in health conditions post sanitation action. However, each health dimension is different and basically cannot be compared in terms of their individual merit; thus, the score for each one needs to be examined and taken into account. For each water supply or sanitation intervention, decision-makers need to establish a proper hierarchy for each separate health dimension.

The overall score comprised a weighted average of the health dimensions scores, to reflect their nature, and was assigned between 1 and 3. These were chosen by the expert team carrying out the study, and were based on observations at the study site, and also information gathered from the interviews. For example, an intervention that focused on environmental education entailed a greater weight in the sociocultural dimension. A decision on the type of system to be implemented gave greater weight to the technological dimension. The decision on the construction of an improvement for water treatment, on the other hand, would carry greater weight for the sanitary dimension. Drilling wells or using rivers for supply would make sense in a region subject to regular extreme droughts, leading to the environmental dimension having greater weight.

## 3. Development of the Dimensions Applied to Health

Seven dimensions were defined when developing the methodology: sanitary, environmental, technological, sociocultural, epidemiological, mental well-being, and economic. These were chosen based on the concept of health adopted by the WHO, “the most complete physical, mental and social well-being” and their concept of water and sanitation, “the control of all factors that may interfere with the most complete physical, mental and social well-being” [3]. They were developed from those proposed in [38], i.e., anthropology, sanitation, epidemiology, and economics, combined with health indicators from [5], including environment and socioeconomic (divided into sociocultural and economic). Mental well-being was also included, as the literature highlights issues such as embarrassment, fear (particularly of assault), lack of privacy, shame, anxiety, and safety, particularly in women and girls, which negatively impacted mental well-being when sanitation was inadequate or lacking, as discussed by Sclara et al. [39].

These are discussed in turn in the following sections.

### 3.1. Sanitary

Sanitation uses environmental surveillance in the identification and monitoring of factors in water, air, and soil that can impact human health, with consequences for the incidence of disease. This, together with epidemiological studies of the incidence and prevalence of diseases and their interrelationship with water and sanitation actions, can be used to control and eliminate risks [40].

### 3.2. Environmental

Society’s impact on the natural environment alters the dynamics of the landscape, modifies its ability to respond, generating a degraded environment, and can lead to disasters. These issues cause concern and impact on sanitation conditions and, consequently, on health.

Pruss-Ustun and Corvalan [41] point out that there are several environmental risk factors that can contribute to the incidence of disease; 24% of diseases in the world can be attributed to environmental risk factors. Data from WHO [40] show that, in 2004, 85 of 102 health problems and injuries were attributed to poor water supply and sanitation. In addition, 24% of illnesses and 23% of premature deaths resulted from exposure to unhealthy environments and unsanitary care.

Disasters can also impact provision of drinking water and can disrupt the sanitation system. According to the UN Office for Disaster Risk Reduction [42], from 1990 to 2014, the most frequent disasters in Brazil were: floods (65.2%), landslides (11.3%), drought (8.7%), storm (7.8%), extreme temperature (3.5%), fire (2.6%) and others (0.9%). Floods caused the greatest number of deaths and homelessness (82.2%), followed by landslides (15.7%). In this context, waterborne diseases are the most frequently observed, followed by those caused by poor water supply and sanitation conditions and food contamination, as well as those caused by changes in the behaviour of disease vectors and infectious agents [10,43]. Therefore, the frequency of occurrence of extreme events is an aspect to be taken into account.

### 3.3. Technological

Technological dimensions are related to the process of selection, conception, and discussion of the technologies to be implemented, preparation of infrastructure projects, and the suitability of technologies adopted. They are linked to other dimensions, since they consider the population’s sociocultural context and verify if the technology is appropriate for the environment in which the sanitation system is to be implemented, from social, cultural, environmental, and economic standpoints.

This approach summarizes to what extent the technological solution used in sanitation projects impacts health. The study of technology is related to a certain context and territory and represents the level of societal development, which is influenced by cultural, political, and economic factors, Murphy [44]. For this reason, the term appropriate technology can be used in specific contexts to mean “technically correct, culturally acceptable and economically viable” [45]. Thus, the implementation of technologies involves risks concerning acceptance and social control; the balance between costs and benefits; in the specificity and scope of its application and in the requirement of behavioural changes in the benefited community [46]. Technology must not only be technical, but the population should be engaged for it to be grounded in the trust of that community [47,48].

Over time, the need to construct water and sanitation systems has caused the emergence of several technological solutions, which needed to be appropriate to benefit each situation and community and to be properly designed for the identified sanitary problems [44]. Therefore, the feasibility of a water and sanitation system depends on several factors, such as: the number of people to be served; aspects related to the community (culture, beliefs, habits, etc.); local environmental conditions; available technology; technical requirements; human, materials, and financial resources [44].

### 3.4. Sociocultural

This is related to the benefits accrued by the population due to the provision of basic sanitation, aimed at understanding the technologies to be used and the way they impact the life and health of the communities involved. Universal access to water and sanitation services is still a challenge to be achieved; the Brazilian National Agency of Water and Basic Sanitation (ANA: https://www.gov.br/ana/pt-br, accessed on 30 September 2022) has to establish targets to achieve it, guaranteed by Brazil’s New Sanitation Legal Framework: Federal Law No. 14.026/2020. For this service to be provided in an equitable way, popular participation, engagement, and awareness are essential factors, which involves communities influencing the creation, implementation, monitoring, and evaluation of public policies [49,50].

Social participation reverses the logic with which the government plans and executes sanitation policies, and the population only receives these services. The population then starts to demand provision of basic sanitation, according to their needs and priorities, and monitor their implementation [11,51]. From this perspective, the sociocultural component seeks to analyse the behaviour, interest, and involvement of the community in relation to water and sanitation, verifying the importance of understanding the relationship between the provision of infrastructure and its impact on health. Thus, similar to the technological aspects, culture, beliefs and habits, as well as the economic aspects of the community, can impact the results of this analysis.

### 3.5. Epidemiological

Poor water and sanitation conditions promote the transmission of biological agents, which may be present in the secretions and excretions of sick individuals or carriers of infectious diseases. Studies show that places with low coverage of basic sanitation services have high incidence rates of diseases such as diarrhoea [52,53], cholera [54], hepatitis [55], intestinal parasitosis [56], and typhoid fever [57], among others. Press-Ustun and Corvalan [41] reviewed data obtained from 145 countries from low- and middle-income countries focused on the prevention of diarrhoea. Their finding highlighted how important improving water and sanitation services is for reducing the burden of disease in these contexts. The great importance in decreasing the incidence rates of these diseases led the National Health Foundation (FUNASA) to classify them as Diseases Related to Inadequate Environmental Sanitation (DRSAI) [58] based on the proposed classification of diseases by Cairncross and Feachem [59] and Mara and Feachem [60].

These diseases have been divided into five major groups according to their form of transmission. They are: (a) faeco-oral transmission diseases (diarrhoea, enteric fevers, hepatitis A); (b) vector-borne diseases (Dengue, yellow fever, leishmaniasis, lymphatic filariasis, malaria, Chagas’ disease); (c) diseases transmitted through contact with water (schistosomiasis, leptospirosis); (d) hygiene-related diseases (eye disease, trachoma, conjunctivitis, skin diseases, superficial mycoses); and (e) geo-helminths and teniases (helminthiasis, teniasis) [58].

The presence of these diseases makes it possible to visualise the precariousness of local basic sanitation systems and whether they constitute a risk to the population, especially the poorest who live in unhealthy conditions. This was shown in a study conducted between 2000 and 2010 [61], which aimed to understand the spatial behaviour of DRSAI throughout Brazil. To do so, they applied the Moran Index to measure the intensity of spatial autocorrelation of each Brazilian municipality and their neighbours in order to determine the relationship between four socioeconomic variables: Municipal Gross Domestic Product per capita; percentage of people with access to water supply, with sanitary sewerage and with rubbish collection and average hospitalisation rates between 2000 and 2010. The study found a negative correlation between rates of hospitalisation and the provision of sanitation services [61].

### 3.6. Mental Well-Being

Mental well-being is an important part of overall health [3]; inadequate sanitation can exert harmful effects on individual and community well-being, and thus it forms a key part of the HIA. The impacts on mental health of inadequate sanitation cause suffering, anxiety, stress, and even depression. A study by Sclar et al., [39], used aspects related to mental health and well-being, such as dignity, privacy, shame, embarrassment, anxiety, fear, violence, and safety to make a broader assessment of the impacts of sanitation on health. These authors concluded that lack of sanitation has a great influence on privacy and safety, aspects that influence anxiety, shame, and embarrassment, especially of women and girls, demonstrating that inadequate sanitation impacts mental health.

A study by Caruso et al., [62] in Sierra Leone, Africa, carried out in eight public schools in two communities with adolescent girls, assessed factors influencing hygiene during menstrual periods in schools. Although all schools had toilet facilities, the quality among them varied. The girls indicated that they did not like to use the school toilets because they had inadequate facilities and smelt. There was also a lack of privacy because there was no separation of toilets by gender. Aspects such as exposure of the body, lack of privacy, and violence were addressed. It was found that during the menstrual period, girls felt greater stigmatisation and marginalisation, irritation, anxiety, and distraction. There were also negative impacts on school attendance.

### 3.7. Economic

The economic component examines costs incurred due to the absence of sanitation infrastructure. Since 2000, the UN MDGs followed by the SDGs have been committed to increasing the proportion of the population with permanent and sustainable access to drinking water and sanitation. A document entitled *Progress on household drinking water, sanitation and hygiene 2000–2020: five years into the SDGs*, by WHO and the United Nations Children’s Fund (UNICEF) [63], highlights that for every dollar spent on the provision of safe drinking water and adequate sanitation, there is an average global return of USD 2.0 and USD 5.5 in terms of health improvement, respectively [64].

Evaluation of the impacts of poor sanitation was conducted by the Lixil Group Corporation in 2016 [65]. Four variables were used in their analysis: premature infant mortality, lost productivity, health expenditure, and value of time lost due to poor sanitation. This study found that there were four regions most affected: Asia and the Pacific, with a cost of USD 172.3 billion, followed by Latin America and the Caribbean (USD 22.2 billion), Africa (USD 19.3 billion) and Eastern Europe, the Middle East and the former USSR (USD 9 billion). The largest expenditure was on premature infant mortality, which accounted for around 50% of the costs in all regions.

From the questions raised in this study around the economic components of water supply, the indicators which would need to be examined include: water and energy consumption and spending on water, energy and health. For sewage treatment, energy consumption to collect and treat sewage, the sewage tariff (included in the water tariff), health expenditure, and the loss of work or absenteeism from school due to sewage-related illness all need to be considered.

## 4. The Development of Indicators for Each Health Dimension

The indicators developed were mainly a combination of those identified by publications such as [5,38] as well as by the fieldwork strategy and expertise of the team as detailed in Section 2. As suggested by [34], many indicators were site-specific, in other words, they were developed “relevant to the proposal in question” (page 12). A WHO database of “Health Indicators” for Brazil (https://data.humdata.org/dataset/who-data-for-brazil?#, accessed on 30 September 2022) concentrated on the following: Disability-adjusted Life Years (per 100,000), distribution of years of life lost by major cause, adult mortality rate (15–60 years, per 1000), deaths per 1000 live births, causes of children’s death <5 years (%), number of deaths. None of these indicators were relevant to the context of a school, requiring specific indicators to be developed as described below.

Indicators associated with the sanitary dimension focused on water quality, and the analysis of specific determinands, which were undertaken during the site visit. The quality of water for human consumption should be monitored in the supply system, distribution networks, reservoirs, or surface sources; this includes operation and maintenance, including at the water treatment plant. Since 1990, the National Program for the Surveillance of the Quality of Water for Human Consumption monitors the quality of drinking water, guided by Consolidation Ordinance GM/MS nº888 [66], and the National Guideline for the Sampling Plan of the Surveillance of the Quality of Water for Human Consumption [67] with samples collected and evaluated for the specific parameters identified in the indicators [68]. The frequency of this analysis was assessed by interaction with the school respondents, since the Standard of Water Potability recommends it is undertaken every 6 months. Food safety was also chosen, since the case study was carried out in a school, and it is important to have potable water (i.e., in terms of quality) for the preparation of school meals. This was determined during the survey by perceptions of the respondents.

The environmental indicators concentrated on water quantity provision, as the case study was in a Brazilian semiarid region, i.e., what quantity was delivered, how frequently, if it was sufficient or not and why. The frequency of extreme events was also important, such as time without rainfall leading to drought. Food preparation can be impacted by drought if there is insufficient water to prepare the food. Much of the information in relation to these indicators was obtained during the school survey and observations during the field visit. Climate issues were obtained from Internet searches such as rainfall in the research location.

Indicators related to the technological dimension were selected to evaluate the existing water supply system at the school. The safest source of supply would be the public network [63], but in rural areas of Brazil, there is generally no connection to the network. Therefore, wells or water tankers can be used, which may not be safe. A further indicator was how water was stored, and if this was in a container, what material it was made of and how often it was cleaned. The best would be tanks made of polyethylene, although many rural schools have rainwater reservoirs, which need to be maintained regularly if present, to ensure cleanliness. The quality of potable supplies is cross-referenced with the chemical analyses conducted in the Sanitary dimension, as well as maintenance of the supply infrastructure, such as changing the drinking fountain filter to ensure there is sufficient-quality water in the distribution system, and at the point of consumption. Water quantity was related to assessments carried out in the Environmental dimension, including water consumption (gained from perception of the respondents) and number of distribution points (observation during the field visit).

In the sociocultural dimension, the proposed indicators covered habits and customs, such as the amount of water ingested daily, habits such as frequency of hand washing, and the washing of fruit and vegetables before eating. An assessment needed to be made of any awareness of the need for water rationing with educational sessions in the school about the importance of water, particularly in terms of hygiene and associated water-related disease. Much of this information was gained from the respondents during the field visit.

For the epidemiological dimension, the focus was on the symptoms and diagnosis of water-related disease such as cholera, verminosis, typhoid fever, gastroenteritis, and leptospirosis. The symptoms were separated from the actual disease, since a diagnosis has to be made by a qualified medical doctor, whereas the symptoms were canvassed from the school community and included incidences of diarrhoea, sickness, headache, etc., as listed below; toothache was also included, as it is related to oral hygiene, the amount of caries, and tooth brushing habits. In semi-arid areas, the likelihood is that water from wells can be brackish and if it is used for drinking, may lead to hypertension, incidences of which needed to be assessed.

Mental health can be adversely affected by a lack of water [69], leading to difficulties with concentration and memory, in addition to overall fatigue as well as mental fatigue. It may also cause irritability, depression, and apathy, hence the importance of the indicators in this dimension, which can lead to absenteeism from work and school, potentially leading to job losses. Thus, the mental well-being indicators reflected issues associated with provision of an adequate supply of water and were related to the perceptions of the respondents.

Indicators for the economic dimension were identified due to their association with the cost of providing improvements to the water supply, and thus were structured around a before/after scenario for water, energy supply, and incidence of disease. Often, schools in rural areas of Brazil rely on water deliveries by tanker, which is expensive; if the implementation of a new system is cheaper, the school will save money. However, it is possible that other expenses would be incurred, such as energy, and thus the “after” scenario in the HIA would be able to indicate this. Reduction in expenses due to water-related disease, particularly in the event of hospitalisation, can be substantial, and can also be associated with lost income and livelihoods. Information related to water and energy expenditure could be obtained from the municipal education offices, through the analysis of water and energy bills, whereas health expenditure data can be obtained via the perceptions of the school community.

Each indicator received a grade from 0 to 1 in order to compute the average grade for each health dimension, and so produce the overall score for health. The grade for the physical and chemical parameters indicate if they conform to legal standards (i.e., one) or zero if they do not conform. For other indicators, different evaluation tools will be used, depending on the nature of the indicator. For each of those evaluation tools, an example is given, but the tool is not limited to the given example. In Table 2 and Table 3, each indicator is related to its relevant evaluation tool (a, b, c, or d):

a. Measuring study—water samples will be collected at the main points of the water supply system.

b. Observational study—the behaviour of water and sanitation facilities will be evaluated and verified to determine whether there is significant damage to the structure of the system and whether the system is working properly.

c. Perception study—aspects of reality will be determined, applying questionnaires in order to raise objective and subjective data on the community affected by the absence of water and sanitation systems.

d. Survey study—of the health data of the community will be carried out, aimed at identifying the main symptoms and diseases.

Due to the different focus of the two kinds of interventions, indicators have been grouped separately for water supply and sanitation.

### 4.1. Indicators Associated with Water Supply Dimensions

#### 4.1.1. Sanitary

- Physical parameters of water quality (temperature, colour, and turbidity).
- Physical parameters for perception of water quality (taste, odour, colour); these may affect the approval of the water for human consumption.
- Chemical water-quality parameters (pH, total and free residual chlorine).
- Microbiological water-quality parameters (total coliforms and faecal coliforms, Escherichia coli). It should be noted that there are many types of biological agents. Some are important in the transformation processes of organic matter in biogeochemical cycles, but others are responsible for causing disease and generating health concerns. For this type of analysis, the most important micro-organisms are the coliform bacteria, which are associated with water-borne disease [69].
- Frequency of water analysis.
- Food safety—related to water quality in food preparation.

#### 4.1.2. Environmental

- Frequency of extreme events.
- Frequency of supply.
- Quantity of water/source supply.
- Water uses: how extreme events (drought or flooding) affect water uses.
- Impact of extreme events on food—related to water quantity during food preparation and types of vegetables and fruits more resistant to extreme events such as drought.

#### 4.1.3. Technological

As water supply is linked to the existing community supply system, all of the process needs to be monitored.

- Type of source (public network, well, water tanker).
- Type of storage (water tank, cistern).
- Material the storage container is made of (polyethylene, fibreglass, metal, asbestos, cement).
- Frequency of water tank cleaning.
- Types of treatment (filtration, water boiling, chlorination or other disinfection process such as ozone, clay filter, activated carbon filter, or even water-treatment plant, i.e., complete treatment with: coagulation, decantation, filtration, and chlorination)
- Distribution points (drinking fountains, kitchen taps, washbasins, showers, etc.).
- Consumption points (kitchen, drinking fountain, etc).
- Operation and maintenance (how the operation is carried out: does the water arrive every day and every hour, or is there intermittent supply?), periodicity of maintenance (normally every 3 months to change the filter, clean the water tank, etc.).

#### 4.1.4. Sociocultural

- Habits and customs (amount of water taken inside a household/ building, washing hands, washing fruit and vegetables, and how this is influenced by the source of supply (public mains, well, etc.))
- Quantity of water ingested.
- Hygiene habits.
- Rational use of water awareness.
- Raising awareness on the use and importance of water (if there are lectures or information on the importance of water conservation and the role of each person).
- Community interest.
- Educational and information, social mechanisms (programmes in schools, advertising, radio and television programmes, and projects).
- Awareness of waterborne diseases.

#### 4.1.5. Epidemiological

- Symptom of diseases (diarrhoea, bloody diarrhoea, yellow skin and/or eyes, red eyes, fever with chills, joint pain, headache, abdominal pain, intestinal pain, lack of appetite, nausea, and/or vomiting and toothache).
- Incidence rates of waterborne diseases (cholera, leptospirosis, verminosis in general, amoebiasis, typhoid or paratyphoid fever, giardiasis or cryptosporidiosis, infectious hepatitis, gastroenteritis, leptospirosis, kidney diseases, hypertension, dental caries, and gingivitis) based on hospitalisation and mortality rates for waterborne diseases.

#### 4.1.6. Mental Well-Being

- Depression (due to lack of water).
- Children’s concentration.
- Emotional and behavioural changes.
- Absenteeism from work and school.
- School learning abilities.
- Incidence rates of mental health-related diseases.

#### 4.1.7. Economic

- Water consumption expenditure.
- Energy consumption expenditure.
- Disease expenditure.

The dimensions and indicators for water supply provision are summarised in Table 2.

### 4.2. Indicators Associated with Sanitation Dimensions (Lack of/Inadequate Sewage System)

#### 4.2.1. Sanitary

The indicators to assess water contamination include:

- Physical parameters of water quality (temperature, taste, odour, colour, turbidity, total solids (suspended and dissolved)).
- Chemical water-quality parameters (pH, alkalinity, acidity, hardness, dissolved oxygen, chemical oxygen demand, biochemical oxygen demand, nitrogen series (ammonium ion nitrite ion, nitrate ion), phosphorus, iron and manganese, micropollutants (heavy metals eg arsenic, cadmium, chromium, copper, lead, mercury, nickel, silver, zinc, cyanides and fluoride), total chlorine, and free residual chlorine.
- Microbiological water-quality parameters (total coliforms and faecal coliforms, Escherichia coli).
- Frequency of water analysis.
- Food safety—related to water quality in food preparation.

#### 4.2.2. Environmental

Due to extreme events (for example, drought or flood):

- Change in volume reduced due to an extreme event.
- Impact on sewage collection.
- Impact on the sewage treatment system.

#### 4.2.3. Technological

- Individual or collective solution for sewage (septic tank or collection network).
- Treatment system adopted (primary, secondary, or tertiary treatment of sewage).
- Final disposal adopted (drying bed, incineration of sewage sludge).
- Operation and maintenance (necessary precautions for the operation of the system, both for the collection network and the treatment system, the need to dispose of the sludge, care with gas leaks (e.g., from a biodigester), etc.).

#### 4.2.4. Sociocultural

- Habits regarding the use of sanitary facilities (how the toilet is cleaned, what is discarded in it such as absorbent pads, etc.) if there is a toilet or only a hole, etc.
- Raise awareness of the population regarding the disposal of sludge.
- Population’s knowledge about reuse of urine and faeces; use of biogas produced from sewage.
- Lectures and educational events, e.g., radio or television programmes, projects using sewage.

#### 4.2.5. Epidemiological

- Incidence rates of diseases caused by inadequate sanitation.
- Hospitalisation rates for diseases caused by inadequate sanitation.
- Mortality rates from diseases caused by inadequate sanitation.

#### 4.2.6. Mental Well-Being

- Depression (due to lack of/inadequate sewage management).
- Stress (due to lack of/ inadequate sewage management).
- Lack of concentration.
- Emotional and behavioural changes due to the type of solution (e.g., women going into the toilet, issues around violence, insecurity, embarrassment).
- Incidence rates of mental-health-related diseases.
- Absenteeism from work/school.
- Changes in school learning.
- Economic impacts.
- Energy consumption spent on sewage collection and treatment.
- The sewage tariff.
- Health expenditure due to diseases caused by sewage.
- Loss of work or absence from school due to illness caused by sewage.

The dimensions and indicators for sanitation are summarised in Table 3.

## 5. Results of a Case Study Applying the HIA Methodology

The application of the HIA methodology was carried out in a case study based in a school located in the Brazilian north-eastern semi-arid region, where there is a deficiency in water supply. In this school, equipment donated by the Israeli government was to be implemented, which extracts water from humid air, therefore improving the quality of water consumed in the school. The HIA methodology will be used to assess the health impact before and after the deployment of the equipment. Thus, health conditions were assessed before the deployment of the equipment to establish baseline. The evaluation of the health impact after the deployment of the equipment will be carried out at the end of 2022.

Field data collection included 4 teachers/educational coordinators, 2 general service assistants (GSA: these are people who work in the kitchens and also have cleaning duties in the school) and 19 students.

In terms of the specific case study, water supply was included as applied to a sanitary intervention in a school, whose dimensions were: sanitary (water quality), environmental (water quantity), technological (the water supply system itself), epidemiological (impact on health due to water-delivery diseases), mental well-being (diseases due to lack of water), sociocultural (habits of the community), and economic factors (financial issues).

The weights of the dimensions were assigned from 1 to 3, based on the relevance of each dimension in relation to the intervention carried out. The weightings were chosen based on observation, perceptions of the individuals in the study, and the expertise of the team who carried out the survey. As this was in a school, the weightings were also site specific, and included information gathered during the site visit. The dimensions weighted 3 were: sanitary, epidemiological, mental well-being and technological because these were central to sanitation and their impacts on health. The environmental dimension was assigned a weight of two because it refers to the amount of water, which also influences disease incidence. The sociocultural and economic dimensions were assigned a weight of one because, despite their importance, they are not directly related to water quality. The score for each indicator varied from 0 to 1, always focused on the best health condition. Table 4, Table 5, Table 6, Table 7, Table 8 and Table 9 summarise the results of the surveys carried out for each dimension and its indicators. Tables showing the calculations underlying the scores are provided in Supplementary Material S2.

In terms of the Economic dimension, during the site visit, it was found that the average monthly energy consumption of the school was 421kwh and that the school is supplied by a 5000-litre water truck every 15 to 20 days. However, it was not possible to score this dimension, since the school budget was not known, and therefore neither were the costs in relation to the budget. The Secretary of Education has been asked for information so that this dimension can be calculated. The calculation of the current overall HIA is a preliminary assessment; it is likely to change once a score for the Economic dimension can be included.

This overall result, where the school is at 46% on the health assessment, shows that there are improvements that need to be made, and therefore there is a high likelihood that the intervention will provide a major boost to this rate. Taking each dimension separately, however, illustrates the scale in terms of their impacts on health, as shown in Table 10, whereby the lowest scores were obtained by sociocultural, mental well-being and environmental dimensions, and the higher scores by epidemiological, sanitary, and technological dimensions. It must be noted that these scores are associated with some indicators which have been estimated, and thus may reflect a certain amount of imprecision in the health dimension of the study community. To contextualise these results, as stated by PAHO/WHO [5] (page 6): “Measuring dimensions of health in a population requires estimations, and therefore there is a certain degree of imprecision.” “Every health indicator is an estimate (a measurement with some degree of imprecision) of a given health dimension in a target population.” Additionally, as [29] (page 42) states: “HIA does not try to uncover absolute and incontrovertible truths.”

## 6. Discussion

This paper presents the details of constructing a HIA methodology based on sanitation actions and their potential to impact health, specifically in a Brazilian context, and applied to the situation of a school in north-east of the country, where sanitation is inadequate. The purpose of developing the HIA methodology was to assess any improvements in the health of a population as a result of the implementation of a water supply or sanitation programme in order to address inadequate sanitation which “reduces human well-being, social and economic development” WHO [11]. This relationship, although obvious, has not been sufficiently substantiated until now. To this end, the methodology took as its starting point the concept of health established by PAHO/ WHO (2018) [5] for its view of health in different dimensions. Whilst HIAs have been used to assess other impacts (e.g., transport [70]; climate change [71]) and in other contexts [35], this is the first time such an approach has been taken in Brazil and the first time health has been considered in terms of seven dimensions to provide a more comprehensive approach than is usually taken.

The indicators assigned to each dimension allowed an assessment to be made of the extent to which health benefits from various water supply or sanitation programmes could be identified, monitored, evaluated, noted, and acted upon. It should be noted that these indicators were identified based on conceptual and operational understanding of a provisional nature during the preliminary field visit, and thus will be subject to modification and improvement with subsequent visits. This reinforces the need for constant feedback throughout the development of the work and evaluation process, in which case studies are fundamental to development of the strategy.

Harris-Roxas et al., [34] evaluated the possibilities of HIA from the perspectives of its strengths, weaknesses, opportunities, and threats. In terms of the strengths of the proposed HIA, it provides an instrument or tool that government agencies can use to evaluate public sanitation and environmental policies, programmes, and actions. In the Brazilian context, its environmental impact assessment (EIA) also contains an evaluation of health outcomes in terms of the production of disease only. Whilst the proposed HIA includes epidemiology, it goes beyond just considering this one dimension, as is the case in the Brazilian EIA, but provides an assessment of a further six, enabling a comprehensive review of the circumstances both before and after introduction of a sanitation action, or introduction of sanitation programmes. It also provides the opportunity to identify specific dimensions which require extra attention. In this case, as is shown in Table 10, sociocultural, mental well-being, and environmental dimensions would need more effort than epidemiological, sanitary, and technological dimensions. These lower health scores represent the urgency of any intervention, whereas the higher scores indicate that the community is able to manage adequate water supplies and sanitation and that improvement is less of a priority. With the lack of other studies using HIA to assess the efficacy of water and sanitation interventions, it is not easy to contextualise an overall score of 46%. However, as this reflects the current situation, before application of an intervention to improve drinking water quality, the outcome indicates substantial room for improvement. It is difficult to predict what the overall score could be, or the effect on individual dimensions at the follow-up visits to assess the sanitation intervention. However, the scores should change to reflect any positive or negative impacts and will give an overview, particularly on health, of the effectiveness of the intervention in improving health and well-being. Any new intervention, even at the same site, but certainly at different ones, would require a further baseline to be established in order to effectively assess its impacts on the health of the community.

It is important to establish the specific parameters which determine the focus of the HIA [34]. In this specific case, it was to produce a broad HIA which included different dimensions, not only epidemiology, and which has the potential to be applicable in other countries with similar issues around inadequate sanitation and health impacts. A further strength is in the ability to target decision-making which is value for money. At many levels, national and international, and the individual school level, financial resources are scarce, thus the benefit of using a comprehensive HIA is the ability to be able to select appropriate water supply and sanitation programmes, in the context of their health benefits, as well as being able to obtain the necessary support from both the social and political sectors. This methodology can additionally support the process of environmental surveillance, providing evidence of negative impacts, not only on health, but also on the surrounding environment and hence on quality of life.

In terms of weaknesses, [34] highlights the complexity around the scoring of different types of impact; the current study utilised perceptions of the school community and observation as the basis of the study (Section 2). It also illustrates the methodology and the preliminary investigation before use of the intervention. In a follow-up, once the sanitary action is in use, the HIA will be applied once more. A further weakness in rolling out such a methodology would be in any financial and human resources required, since Brazilian municipalities are short of both, particularly trained technicians. Therefore, if HIA became public policy whereby each municipality had to undertake sanitation work, in all likelihood, the Ministry of Health would charge for work. However, as is shown by Ferreira et al. [26], the increased efficiency of Brazil’s sanitation system could go some way towards offsetting the costs of sanitation actions by monetising the reductions in hospitalisation due to water- and sanitation-related diseases. Thus, average numbers not requiring hospital treatment due to investment in sanitation could be as much as 157 thousand per BRL 100 million with a potential 26 thousand per BRL 100 million due to investment in drinking water supplies. Such substantial reductions in hospital admissions could be promoted by relatively little financial outlay to improve provision of adequate sanitation and water supplies.

Opportunities of the HIA revolve around its flexibility in that it provides the opportunity to undertake an assessment of the seven dimensions and can identify areas in which more urgent action is needed. It is, therefore, an opportunity to evaluate the situation before and after the application of a sanitation action and make a judgement of the best approach to take once the weaker dimensions have been identified. The method also enables engagement of the community in discussions around their perceptions of the situation, enabling contextualisation and the employment of a more site-specific strategy.

The final perspective of [34] is that of threat, and in this case, it is the fact that government organisations do not necessarily engage with one another; for example, the health sector is not involved with the planning of other sectors, such as provision of sanitation. This HIA methodology covers many government sectors and organisations, and thus encourages dialogue between them to support joint and integrated planning efforts.

Once the overall health score has been defined, the weights assigned to each of the health dimensions depending on the type of intervention can be used to define the work to be carried out. Another application is the verification of the health impact caused by the intervention, in order to support and defend its implementation for legislators, politicians, and society at large.

This, therefore, has the potential to be a valuable tool to support decision making with regard to investment in water supply and sanitation programmes by providing a comprehensive set of indicators related to health dimensions in terms of sanitation actions. This firstly assesses any issues with regard to sanitation provision in a specific context, and secondly provides a numerical measure for any improvements gained by managing inadequate sanitation.

The limitations of the study include the fact that it is presenting the development of the methodology, and the case study illustrating its use is preliminary and provides information to establish baseline only, i.e., before an intervention is applied to improve a situation where sanitation is inadequate. The Economic dimension has not been addressed due to lack of information on the school budget; however, this will be accessed during further field visits. A further limitation is that it is applied in only one school; however, two more evaluations in the same school are planned, with testing of the methodology followed up in three more schools, in other states, for which permission has been granted. This will enable the assessment of any improvement in individual dimension scores, as well as the overall HIA score. Currently, a further limitation is that the methodology is only used in the Brazilian context, but once the HIA has been trialled, it will be assessed for its utility in other countries with similar issues of lack (or inadequate provision) of sanitation.

## 7. Conclusions

The rationale for proposing a methodology for an integrated HIA focused on Brazil is that for many years, the measurement of the effectiveness of public policies related to water and sanitation has been by accounting for the benefited population or the financial investment, or even through the efficiency of the system itself. The greatest impact of improved water and sanitation infrastructure is on the improvement in the population’s health, not simply on the absence of disease. This paper, therefore, proposes a methodology that can be used as a tool to discuss with those who will benefit about the type of intervention in water and sanitation to be used, and then to measure the consequences of the intervention itself on health, but also to enable policymakers and legislators to engage with the process. The proposed methodology has only been used in Brazil thus far, but has the potential to be used in other developing countries with similar issues around inadequate sanitation and its impacts on health.

## Figures and Tables

**Table 1:** Percentage of households with a water supply and sanitary sewage connection from Brazil’s MIC3 (1996) [18] and from IBGE 2010 (https://sidra.ibge.gov.br/, accessed on 30 September 2022). HH = household.

	Urban	Rural	South	South-East	North-East	North	Mid West	Total
**% HH with a water supply in the house/on the land**	84.3	24.9	78.9	83.6	66.9	73.7	71.3	72.7
**% HH with a bathroom and sewage connected to a collection network**	50.5	6.3	31.9	61.6	14.7	5.6	29.2	41.7
**Population (x 1000) 1996**	128,897	35,718	23,356	63,000	45,540	11,410	10,465	157,871

**Table 2:** A summary of the components and indicators related to water supply used in the HIA showing impacts on health. Where (a.) to (d.) relate to the evaluation tools given in Section 4.

Dimensions Related to Water Supply Provision						
Sanitary health (a.) and (c.)	Environmental (b.) and (c.)	Technological (b.) and (c.)	Socio-cultural (c.)	Epidemiological (c.) and (d.)	Mental well-being (c.)	Economic (c.) and (d.)
**Indicators**						
Physical water quality parameters	Frequency of supply	Type of source	Habits and customs	Incidence rates of waterborne diseases	School attendance	Water consumption
Chemical water quality parameters	Quantity of water/source of supply	Storage type	Hygiene habits	Hospitalisation rates for waterborne diseases	School dropouts	Energy consumption
Microbiological water quality parameters	Water use	Types of treatment	Raising awareness of the use and importance of water	Mortality rates from waterborne diseases	Absenteeism	Water expenditure
Frequency of water analysis		Distribution points	Community interest		Emotional and behavioural changes	Energy expenditure
Cleaning of the storage system and frequency		Operation and maintenance	Educational and information		Changes in school learning	Health expenditure
Food safety			Socialisation		Incidence rates of mental health-related diseases	
			Quantity of water ingested and how			

**Table 3:** A summary of the dimensions and indicators related to the lack of, or inadequate management of, sanitation used in the HIA showing impacts on health. Where (a.) to (d.) relate to the evaluation tools given in Section 4.

Dimensions Related to Lack of/Inadequate Sanitation						
Sanitary health (a.) and (c.)	Environmental (b.) and (c.)	Technological (b.) and (c.)	Socio-cultural (c.)	Epidemiological (c.) and (d.)	Mental well-being (c.)	Economic (c.) and (d.)
**Indicators**						
Sewage system (mains or septic tank)	Due to extreme events:	Individual or collective sewage solution	Habits and customs regarding the use of toilet facilities	Rates of disease caused by inadequate sanitation	School attendance	Energy consumption spent on sewage collection and treatment
Treatment	Change in volume	Treatment system adopted	Raising the population’s awareness of disposal	Hospitalisation rates for disease caused by inadequate sanitation	School dropouts	Sewage tariff
Final disposal	Impact on sewage collection	Final disposal adopted	Population’s knowledge about reuse of urine and faeces; use of biogas produced from sewage	Mortality rates from disease caused by inadequate sanitation	Absenteeism	Health expenditure due to disease caused by sewage
Leakage	Impact on the sewage treatment system	Operation and maintenance	Lectures and educational events		Emotional and behavioural changes	Loss of work or absence from school due to illness caused by sewage
Interconnection with the water supply system					Changes in school learning	
					Rates of mental health-related disease	

**Table 4:** Results of the survey carried out for the Sanitary Dimension and its indicators. Weighting was 3; where n = total number of respondents, TU = Turbidity Units.

Indicator	Parameter	Water Quality Analysis	Participant Statements Collected from the School Community. Where: N^o^ (%)	Score (0 to 1)
**Physical parameters of water quality**	Colour	Samples were 0 µH, below the Maximum Allowed Value of 15 µH (Brazilian Water Potability Standard: Portaria MS n°2914/2011)	-	1
	Turbidity	Samples were 0.12UT to 0.41UT, below the Maximum Allowed Value, 5.0UT (Brazilian Water Potability Norm Portaria MS n°888/2021)	-	1
	Temperature	Temperature was at a value accepted by the population and found in Brazilian aquatic environments (ie 20–30 °C)	-	1
**Physical parameters for perception of water quality (n = 25)**	Taste (adequate water is tasteless)	-	11 (44%) the water was tasteless.	0.44
	Colour (suitable water is colourless)	-	15 (60%) the water was transparent.	0.60
	Odour (adequate water is odourless)	-	19 (76%) the water had no smell	0.76
**Average physical parameters**				**0.8**
**Chemical parameters for water quality**	pH	Results ranged from 7.4 to 7.7 within the range recommended in the Potability Standard (from 6.0 to 9.0). Portaria|MS No. 2914/2011		1
	Free residual chlorine	Results ranged from 0.06 to 0.08 mg/L indicating residual chlorine below the Potability Standard (0.2 to 2.0 mg/L)		0
**Average chemical parameters**				**0.5**
**Microbiological Parameters**	Total coliforms	Total coliforms were present: these should be absent in 100 ml of water according to The Brazilian Water Potability Standard.		0
	*Escherichia coli*	*E. coli* was absent		1
**Average microbiological parameters**				**0.5**
**Water analysis frequency: The Ordinance on Water Potability, Ministry of Health, Brazil requires annual sampling (n = 6)**		-	1 (17%) indicated once a year	0.17
**Food safety (as related to water quality in food preparation)**		The kitchen water sample was contaminated with total coliform, thus not suitable for food preparation	-	0
**Average overall score =**				0.39

**Table 5:** Results of the survey carried out for the Environmental Dimension and its indicators. Weighting was 2; where n = total number of respondents.

Indicator	Participant Statements Collected from the School Community. Where: No (%)	Observations during the Site Visit	Score (0 to 1)
**Frequency of extreme events (drought) (n = 6)**	1 (17%) of respondents said that there has been no drought *^1^	Annual rainfall index of 641.7 mm with droughts lasting 6 to 8 months. The site is classified as a hot semi-arid climate with a rainfall variation of 250–750 mm per year (Brazil, 2020). As the maximum rainfall in Brazil is 1800 mm per year, 641.7 mm is equivalent to 35.65%	0.263
**Frequency of water supply (4 teachers and 2 GSAs only, n = 6)**	3 (50%) of respondents said that water arrives every day. This question was only asked of 4 teachers and 2 GSAs.	Water supply was not regular. Water in the well was brackish; the school was supplied with a water truck every 15 to 20 days *^2^.	0.25
**Quantity of water**	9 (36%) there is abundant water	-	0.36
**Types of water use (n = 25)** **(n = 2)**	12 (48%) water was used for drinking, washing hands, brushing teeth and flushing toilets, it is also used in the kitchen. 2 (100%) toilet cleaning occurs daily using water and cleaning materials (GSA).	-	0.74
**Impact of drought on food (n = 25)**	7 (28%) the drought does not, or rarely, impacts food	-	0.28
**Impact of drought on cleaning (n = 2)**	2 (100%) drought does not harm the cleanliness of the school (GSA)	-	1
**Average overall score**			**0.48**

*^1^ Documentary evidence of rainfall indices from the area confirmed this. *^2^ This only considered information from the school community and the percentage of people who said that there were daily water deliveries, which is considered to be the best situation for health. However, the site visit found that the water tanker only supplied the school for 15 to 20 days.

**Table 6:** Results of the survey carried out for the Technological Dimension and its indicators. Weighting was 3; where n = total number of respondents.

Indicator	Participant Statements collected from the School Community where: N^o^ (%)	Observations during the Site Visit	Score (0 to 1)
**Water source type (n = 25)**	6 (24%): water was supplied from a well, considered a safe source according to WHO. 5 (20%): from a pipe from the street, 4 (16%): delivered by water tanker.	The well water was brackish and supplies the kitchen and lavatories. There was no frequent water supply. No water was supplied from the street pipe. The tanker supplied fresh water, distributed via the school’s drinking fountain.	0.30 *^1^
**Water storage type (n = 25)**	7 (28%): cisterns and water tanks. 13 (52%): water tanks, 1 (4%) cistern.	There was a cistern on the ground and a raised water tank.	0.92 *^2^
**Maintenance of the water supply system (teachers only; n = 4)**	4 (100%): the water tank had a lid.	The supported cistern had a lid, but the water tank was uncovered *^3^.	0.5
**Frequency of water tank cleaning (n = 6)**	4 (66%): water tank hygiene was carried out at intervals of between 1 and 6 months	-	0.66
**Maintenance of equipment used for water treatment (n = 6)**	3 (50%): the filter candle was changed at intervals of between 6 months to 1 year.	-	0.50
**Type of material used for storage (verified by colour)** **n = 25**	11 (44%): blue (plastic), 5 (20%): white box, 2 (8%): cement, 1 (4%): grey.	The two boxes were made of concrete, with the cistern painted white *^4^	0.505
**Water distribution points (n = 25)**	3 (12%): water was distributed via a drinking fountain, hand washing sink, the shower, toilet, kitchen sink and tank.	The places where water was delivered were verified, ie the drinking fountain, sinks, shower, toilet, kitchen sink and tank. However, there was no filter on the kitchen tap.	0.56
**Water consumption points (n = 25)**	14 (56%): drinking fountain, 6 (24%): kitchen tap with filter *^5^	-	0.80
**Treatment, GSA only (n = 2)**	2(100%): washing was performed whenever the candle was dirty or every month		1
**Average overall score**			**0.64**

^*1^ Average of sum of sources and observations; ^*2^ average of all perceptions and observations. ^*3^ was considered zero since the tank had no lid. A score of 0 reflects hygiene of the water tank and 1 for treatment, as the filter candle was frequently changed. *^4^ all answers were added together because the reservoirs were concrete, one of which was painted white. *^5^ There was both a water dispenser and a kitchen tap, the two responses were added together.

**Table 7:** Results of the survey carried out for the Epidemiological Dimension and its indicators. Weighting was 3, where n = total number of respondents.

Indicator		Information Collected from the School Community: N^o^ (%) of Respondents not Experienced Symptoms, Infections, Disease or Condition	Health Data (for the Municipality)	Score (0 to 1)
**Symptom** **N = 25**	Diarrhoea (stomach ache)	6 (24%)		0.24
	Bloody diarrhoea	13 (60%)		0.60
	Yellowish skin and/or eyes	18 (72%)		0.72
	Red eyes	12 (48%)		0.48
	Fever with chills	10 (40%)		0.40
	Joint pain	8 (32%)		0.32
	Headaches	2 (8%)		0.08
	Abdominal pain	7 (28%)		0.28
	Intestinal pain	11 (52%)		0.52
	Lack of appetite	9 (36%)		0.36
	Nausea and/or vomiting	11 (44%)		0.44
	Toothache	5 (20%)		0.20
**Average symptoms**				**0.39**
**Disease diagnosis** **N = 25**	Diarrhoea *^1^	6 (24%)	(a) 8(2019); 6(2020) and 1(2021) (b) 2(2020)	0.12
	Verminosis in general	16 (64%)		0.64
	Amoebiasis	17 (68%)		0.68
	Typhoid or paratyphoid fever	16 (64%)		0.64
	Giardiasis or cryptosporidiosis	17 (68%)	a) 1(2019)	0.34
	Cholera	16 (64%)		0.64
	Kidney disease	19 (76%)		0.76
	Hepatitis, infectious	16 (68%)	c) 1(2021)	0.34
	Gastroenteritis	17 (68%)	(c) 7(2019), 10(2020) and 2(2021) *^2^	0.34
	Leptospirosis	19 (76%)		0.38
	Hypertension	10 (40%)		0.4
	Dental caries	9 (36%)		0.36
	Gingivitis	11 (44%)		0.44
**Average disease diagnosis**				0.47
**Average overall score**				**0.43**

(a) ICD-10 Mortality Monitoring Panel; (b) Mortality Information System (SIM). (c) Hospital information system. ^*1^ Population in the municipality. ^*2^ Such as infectious intestinal diseases.

**Table 8:** Results of the survey carried out for the mental well-being Dimension and its indicators. Weighting was 3; where n = total number of respondents.

Indicator	Participant Statements Collected from the School Community. Where: No (%)	Score (0 to 1)
**School dropout (n = 4)**	4 (100%) no school dropout due to drought	0 *^1^
**Absenteeism from work and school (n = 6)** **(n = 19)**	2 (33%) no work missed due to drought. 12 (57%) no student absenteeism	0.45
**Depression (n = 25)**	11 (44%) no cases of depression	0.44
**Children’s learning (n = 25)**	5 (22%) no change in learning ability	0.22
**Children’s concentration (n = 4)**	1 (25%) students were not affected during drought	0.25
**Behavioural changes (n = 25)**	10 (40%) no behavioural changes during drought	0.40
**Average overall score**		**0.29**

*^1^ = Was considered zero in terms of school absence as all teachers said that there was none due to drought.

**Table 9:** Results of the survey carried out for the Sociocultural Dimension and its indicators. Weighting was 1; where n = total number of respondents.

**Indicator**	**Participant Statements Collected from the School Community. Where: No (%)**	**Score (0 to 1)**
**Daily amount of water intake (n = 25)**	6 (24%) 2 litres of water per day consumed as recommended by WHO	0.24
**Hygiene habits (brushing teeth, washing fruit and vegetables (n = 19)** **For 2 GSA only: wash eggs, wash hands, wear gloves and wear closed shoes when cleaning at school** **(n = 2)**	7 (38%) of students brush their teeth at school 12 (57%) wash fruit, vegetables and greens 2 (100%) GSA wash eggs 2 (100%) GSA wash their hands frequently 2 (100%) GSA wear gloves, 2 (100%) GSA wear closed shoes when cleaning and 2 (100%) GSA wear a cap to cover their hair when working in the kitchen	0.85 *^1^
**Use of personal protective equipment (use of gloves and wearing closed shoes) (n = 2)**	2 (100%) use gloves when cleaning and wear closed shoes during cleaning	1
**Rational use of water awareness (n = 25)** **(n = 19)**	22 (88%) ration their water use/turn off taps during and after use. 13 (69%) students use water when brushing their teeth or rinsing afterwards.	0.785 *^2^
**Raising awareness of the importance of water** **(n = 2)**	2 (100%) GSA showed the importance of water treatment as only clean or filtered water is good for drinking, cooking and brushing teeth.	1 *^3^
**Awareness-raising of water (importance of water, know that only clean is to be used, educational events, talking about water in the classroom, knowledge about waterborne disease)**	2 (100%) demonstrated care with water, promptly solving leaks and 2 (100%) know that only clean water is to be used in cooking, drinking and brushing teeth (n = 2) 7 (28%) said that there are educational events in the school (n = 25) 19 (83%) said that teachers talk about water in class (n = 25) 16 (76%) have heard about water borne diseases due to talks in school, on the radio or television (n = 25)	0.88 *^4^
**Average overall score**		**0.83**

*^1^ Combines all respondents’ perceptions. ^*2^ Takes account of responses to both water awareness. ^*3^ This question was only asked of the 2 GSAs. ^*4^ combines all responses. Economic Dimension = Weighted 1.

**Table 10:** Scores and weightings per dimension.

Dimension	Weighting	Initial Score	Final Score
**Socio-Cultural**	1	0.73	0.73
**Mental well-being**	3	0.29	0.87
**Environmental**	2	0.38	0.96
**Sanitary**	3	0.39	1.17
**Epidemiological**	3	0.43	1.29
**Technological**	3	0.64	1.92
**Weighted average**			**6.94/15 = 0.46**

## Data Availability

This research has different types of information. Water-quality data were provided by a locus analysis by a member of our team. Data from the water system of the school were provided by a locus by member of our evaluation team. The perception data were obtained using questionnaires answered by school members (students and employees) who signed informed consent statements. The secondary health data were obtained from the National Survey of Health Information: ICD-10 Mortality Monitoring Panel; Mortality Information System (SIM), and the Hospital information system.

## Supplementary Materials

The following supporting information can be downloaded at: https://www.mdpi.com/article/10.3390/ijerph191912776/s1, Supplementary Material S1: English translation of Brazilian documents; Supplementary Material S2: Tables S4–S9 complete with calculations of the scores.
