# Supplementary material for: Methodology for a Comprehensive Health Impact Assessment in Water Supply and Sanitation Programmes for Brazil
Source: Int J Environ Res Public Health. 2022 Oct 6;19(19):12776. doi: 10.3390/ijerph191912776 (PMC9565092; doi:10.3390/ijerph191912776)
Supplement: Supplementary file 1 [file ijerph-19-12776-s001.zip › ijerph-1884647-supplementary.pdf]

Supplementary Material S1: English translation of Brazilian documents

1. PLANSAB - National Basic Sanitation Plan. Document under review submitted for the consideration of the national health, water resources and environment councils

1. introduction

The original version of the National Sanitation Plan (Plansab), the main instrument sanitation national public policy, was elaborated by the Federal Government in a broad participatory process and in total harmony with the Law n° 11.445/2007 (Law of National Guidelines for Basic for Basic Sanitation). In the first stage of the elaboration of the Plan, the "Pact for Basic Sanitation: more health, quality of life and citizenship", approved by the Council of Cities Council (ConCidades) in July 2008 and approved by the Minister of Cities in December 2008. The document sought to guide the construction of paths and solutions for the universalization of access to basic sanitation and social inclusion and had the purpose of mobilizing various segments of society for the construction of the Plan, as well as their engagement to achieve the proposed objectives and the proposed objectives and goals.

In this context, Ordinance No. 462 of September 24, 2008 established the Interministerial

(GTI), "with the purpose of structuring the strategic project of elaboration of the National Plan for Basic National Plan of Basic Sanitation", being composed by the Ministry of Cities (MCidades) (National Secretariats of Environmental Sanitation - SNSA, of Housing - SNH, of Accessibility and Urban Programs - Snapu, of Transportation and Urban Mobility - Semob and Executive Secretariat of ConCidades); by the Ministry of Environment (MMA) (Secretariat of Water Resources and Urban Urban Environment - SRHU and the National Water Agency - ANA); for the Ministry of Health (MS) (Health Surveillance Secretariat - SVS and the National Health Foundation - Funasa); by the Ministry Integration (MI) (Secretary of Water Infrastructure - SIH and Development Company of the Development Company of the Valleys of São Francisco and Parnaíba - Codevasf) and by the Ministry of Planning , Budget and Management (MPOG). At the same time, the ConCidades instituted the Follow-up Group (GA), formed by representatives of the different segments that made up the Technical Committee for Environmental Sanitation (CTSA).

On August 18, 2009, Decree No. 6,942 established the Brazilian Sanitation Biennium (2009-2010), with the objective to "promote and intensify the formulation and implementation of policies programs and projects related to basic sanitation, in order to guarantee the universalization of the universalization of services" and "consolidate the elaboration process of the National Sanitation Plan". Besides this, the Decree created the Interinstitutional Working Group, in charge of to coordinate the preparation and promote the dissemination of the Plansab during the various stages of its its development, and also to:

- Elaborate the diagnosis of the situation of basic sanitation services in Brazil, to guide the definition of the Plansab's objectives and goals;
- Plan, execute and coordinate the elaboration process of the Plan, in a transparent and participatory way, by means of regional seminars, hearings and public consultations after hearing the National Health, Water Resources and Environment Councils;
- Elaborate the consolidated version of the Plansab and submit it to the consultative appreciation of ConCidades, of the MCidades; and
- Submit the Plansab to the approval of the Minister of State of the Cities.

As a relevant landmark for the materialization of the original version of the Plansab, that, in parallel to its development, a broad study called Panorama of Basic Sanitation in Brazil, developed by three universities: the Federal University Federal University of Minas Gerais (UFMG), the Federal University of Bahia (UFBA) and the Federal University of Rio de Janeiro (UFRJ). The study, consisting of seven volumes originated the main elements for the preliminary version of the Plan, complementing and detailing the document.

The final result obtained reflected the crowning achievement of a participative process, developed for the elaboration of a national plan for the elaboration of a national plan that sought to contemplate a plural vision of the set of social actors involved in the theme of basic sanitation. The original version of

the Plansab was approved, then, by Decree No. 8141/2013 and Inter-ministerial Ordinance No. 571/2013, contemplating an integrated approach to basic sanitation, which includes the four components: drinking water supply, sanitary sewage, urban cleaning and management of solid management, and drainage and management of urban rainwater. As foreseen in legislation, the Plan was conceived with a twenty-year horizon (2014 to 2033), with provision for annual evaluations and reviews every four Years.

The Decree No. 8,141/2013 also established the Inter-institutional Working Group for Monitoring the Implementation of the Plansab (GTI-Plansab), whose members were appointed by Ordinance No. 171, dated April 9, 2014, amended by Ordinance No. 684, dated October 24, 2014, and is composed of representatives of the MCidades, in charge of coordinating it; of the CC/PR; of the MF; MS; MPOG; MMA; MI; Caixa; BNDES; Funasa; ANA; the National Council Health (CNS); National Council of Environment (Conama); National Council of Water Resources (CNRH); and ConCidades. The GTI-Plansab is responsible, among other activities, for appreciation and approval of the Annual Evaluation Reports of Plansab for 2014, 2015 and 2016.

Thus, after the first implementation period of Plansab (2014-2017), the National Sanitation Secretariat of the Ministry of Regional Development (SNS/MDR) proceeded to review the Plan, through the following stages: i) hearing with associations civil society; ii) hearing with representatives of the Federal Government; iii) approval of the initial structure of the review by the iii) approval of the initial review structure by the GTI-Plansab; iv) formation of an internal group at SNS to act in the construction of the preliminary version of the revised Plansab; v) appreciation and approval of the preliminary version of the revised Plansab by the GTI-Plansab; and vi) discussion of the revised version of the Plansab at public hearings and making the document available for public consultation. The next stages include the appreciation of the document by the councils and by the Minister of Regional Development, and finally the forwarding of the draft Decree.

In the development of the review work, the necessary technical approach was combined with the participatory process. Several meetings and hearings were held, with specialists and Federal Government teams, in order to validate the various intermediate stages of the work, to think collectively about planning scenarios, and to share strategic decisions. In addition, six thematic booklets to the thirteen of the original, which deepened the definitions needed to understand and take a position on specific aspects addressed in the Plan's revision.

In addition, in compliance with the legal provisions and in order to guarantee to the population clarification of possible doubts, as well as the possibility of presenting criticisms and suggestions the proposed revision of the Plan, two public hearings were held, one of which was transmitted by the internet. During the public consultation phase, the proposed revision of the Plan was available on the MDR website to receive contributions through an electronic form. A total of 2,653 amendments were received, of which 39% were accepted or partially accepted. The others (61%) were not accepted for several reasons, duly justified, especially for the need to maintain the completeness and coherence of the document as a whole.

This revised version of the Plansab preserves the logic of planning that emphasizes a strategic vision of the future. In this model, the future is not simply a reality designed by the planning team, which seeks to visualize possible contexts, called scenarios, based on the uncertainties and on a solid analysis of the current and past situation. It starts from the premise that it is not possible to predict the future, but only to make forecasts of possibilities, seeking to reduce the risks of the poss ibilities, seeking to reduce the risks of uncertainties and provide tools that facilitate the definition of strategies.

Besides respecting the structure of the original version of the Plansab and in order to maintain the same coherence, this revised version revisited the Panorama of Basic Sanitation in Brazil, which version of the Plan, seeking, among others, to identify planning scenarios more consistent with reality.

There remains the need to ensure the effectiveness of the implementation of the revised Plan, the way it was conceived. The creation of a permanent body in the Federal Government, linked to the MDR, to

exercise strict and continuous monitoring and evaluation of the Plan and of the public policies of basic sanitation at a national level, including the monitoring of the goals, strategies, programs and the occurrence of the scenarios themselves. Thus, it will be possible to identify non-conformities and trigger measures for their correction. In addition, it will be possible to identify the directions taken by the future reality, comparing it with the scenarios and, in case a strong divergence from the reference scenario is verified, the implementation of implementation of adjustments in the Plan, especially during its reviews every four years, as provided by law.

It is necessary to point out that a central concept from the original version of the Plan, referring to structural measures and structuring measures, remains valid. The former correspond to the traditional investments in works, with relevant physical interventions in the territories, for the conformation of the physical infrastructure for drinking water supply, sanitary sewage, urban cleaning and solid waste management, urban cleaning and solid waste management, and drainage and management of urban rainwater. They are evidently necessary to overcome the deficit of coverage by the services and the protection of the protection of the population against epidemiological, health, property and environmental risks. On the other hand, structuring measures are understood as those that provide political and managerial support for the sustainability of service delivery. They are found both in the sphere of management improvement management, in all its dimensions, as well as in the daily and routine improvement of physical infrastructure.

Thus, the revision of the Plansab seeks to maintain the premise of displacing the traditional focus on the hegemony of investments in physical works, to a better balance of these with structuring physical works, to a better balance of these with structuring measures, based on the assumption that the strengthening of actions in structuring measures will ensure growing efficiency, effectiveness and sustainability of investments in structural measures. In the horizon of the Plan, it is projected the gradual substitution of efforts for the implementation of structural measures to others that strongly value the structuring ones.

In the revised Plansab we opted to keep most of the structure adopted in the original version. The document begins with a summary presentation of the legal bases and the guiding principles and guidelines for the development of the Plan. Next, it presentes the situational analysis of basic sanitation in Brazil, composed of studies on deficits, federal programs and actions, investments made, and the political-institutional evaluation of the sector. In the following sections, the three scenarios for the basic sanitation policy are described and the shown the targets per macro-region, for the reference scenario. After that, the projection of the need for investments, by basic sanitation component, to meet the foreseen targets. Subsequently, the strategies and government programs deemed necessary to understood as necessary for the effective, efficient and effective materialization of the Plan. Finally, the structure and procedures for the monitoring, systematic evaluation and revision of the Plan are proposed.

This document, the result of the process described, is the one that is sent to the councils for their appreciation, discussion with a view to later consolidating its final form, in light of the contributions made. Afterwards, the document will be sent to the Minister of Regional Development for appreciation.

2. The National Survey of Basic Sanitation – Water Supply and Sanitary Sewer (PNSB 2017)

The survey has as target population all entities that provide water supply services through the general distribution network and sanitary sewage collection system, offered by the government or by an agent to whom it has delegated the provision. In addition to the Sanitation Supplement - 2017 of the Survey of Basic Municipal Information (MUNIC), answered by city halls, the PNSB 2017 provides technical data on each of the service operation stages, in addition to information on delegation instruments, services' fees, regularity of water supply, among other aspects. The results will be available for the following territorial areas: Brazil, Major Regions and Federation Units and by ranges of population size.

PNSB 2017: Water supply is present in 99.6% of the municipalities, but sewage treatment, in only 60.3%

The percentage of municipalities with water supply by means of a general system hit 99.6% (5,548 municipalities) in 2017, being the system fully at work in 5,517 municipalities, with activities interrupted in 22 and still being implemented in nine municipalities. In 2008, that proportion was 99.4% (5,531). In 2017, supply was suspended for six or more hours in 44.5% (2,454) of the municipalities with active supply services and there was rationing in 20.8% (1,146). Both conditions were most commonly observed in the Northeast Region, where there was intermittent supply in 67.7% of the locations and rationing in 42.5% of them. Of the 22 municipalities without water supply by means of a general system, 13 were in the Northeast, seven in the North and two in the Central West.

There were, in 2017, 59.8 million active household economies (housing units with water supply where there was a bill paid and/or consumption) supplied in the country, an increase of 32% against 2008 (45.3 million). In spite of that, Brazil still had 9.6 million housing units without water supply by means of a general system in 2017. That number of active household economies supplied corresponds to 86.1% of the housing units in the country. There was also a great difference between the Major Regions: 47.6% in the North; 73.4% in the Northeast; 90.9% in the Central West; 93.3% in the South and 97% in the Southeast.

As for treatment, 4,873 (88.3%) of the municipalities with active services had Water Treatment Stations (ETAs) and/or Simplified Treatment Units (UTSs) in operation in 2017. The Central West (97.6%) and the South (97%) had the biggest percentages of municipalities with ETAs and/or UTSs in operation, whereas the Northeast, the lowest (75.8%). Among the localities with active water supply services, 11.7% had no treatment, reaching 24.2% in the Northeast, 21.6% in the North, 4.6% in the Southeast, 3.0% in the South and 2.4% in the Central West.

A total 5.5% of the volume of water distributed in the country is not treated before reaching the population. Considering the volume treated and supplied, 75.1% receives conventional treatment, which encompasses the steps of flocculation, decantation, filtration, desinfection and, occasionally, additional steps. Another 4.2 receive non-conventional treatment (without all those steps); and 20%, only disinfection (and, occasionally, fluoridation and correction of pH).

About 40% of the water was wasted between the entry to the distribution system and the final destination for the user. In 2017, 52.4 million m³/day were obtained (fresh water: 50.98 million m3/day and brackish water 1.47 million m3/day); 45 million m³/day treated, 46.1 million m³/day distributed (being 43.6 million m³/day treated and 2.5 million m³/day not treated) and only 26.6 million m³/day consumed.

In relation to the entities responsible for the execution of the service, in 2008, the state sanitation companies and the municipal authorities were the executors, respectively, in 66.4% and 9.3% of the municipalities, in 2017 these percentages changed to 69.5% and 10.3%. In relation to city governments, private companies and the associations were the executors in 41.6%, 4.5% and 13.1% in 2008; in 2017, were in charge of the service in 35.2%, 3.6% and 11.6% of the municipalities, respectively.

The survey also showed that the coverage of sewage sanitation by data collection changed from 55.2% (3,069 municipalities) in 2008 to 60.3% (3,359) in 2017, and in 3,206 localities the service was active and in 153 being implemented. Nevertheless, in 2,211 municipalities (39.7%), there was no offer of the service. Whereas in the Southeast, the sewage disposal network served 96.5% of the municipalities, in the North that percentage was of only 16.2%.

There were, in 2017, 35.3 million active household economies with sewage disposal (housing units with water supply where there was a bill paid and/or consumption) in the country, an increase of 39.2% in relation to 2008 (25.4 million). Even so, the country still had 34.1 million housing units without treatment by network in 2017. That number of active economies with sewage disposal corresponds to 50.8% in the housing units, a percentage significantly lower to the coverage of water supply. There was also a major change in the Major Regions: 7.4% in the North; 25.4% in the Northeast; 41.6% in the South; 50.8% in the Central West, and 76.7% in the Southeast.

As for treatment, 62.8% of the municipalities with active services (2,013) had Sewage Treatment Stations (ETEs) in operation. The Central West and the South registered the biggest percentages of municipalities with ETEs (94.4% and 71.7%, respectively), whereas the Northeast, the lowest (51.2%). Among the localities with sewage disposal services, 37.2% had no treatment, and reached 48.8% in the Northeast, 37.3% in the Southeast, 30.6% in the North, 28.3% in the South and 5.6% in the Central West.

The volume of sewage treated per day (11.0 million m³) corresponds to 77.1% of the volume of sewage collected, measured or estimated by the service-rendering agencies. In relation to the level of treatment (preliminary, primary, secondary and tertiary, in increasing order of efficiency), 69.8% of the volume treated was of the secondary type (oxidation of the organic charge by means of the action of microorganisms), 21.9% of the tertiary type (removal of pollutants such as nutrients, pathogenic agents, inorganic solids dissolved or in suspension), 5.9% of the primary type (removal of sedimentable floating solids in suspension), 2.4% of only preliminary type of treatment (removal of oil, floating dirt and sand).

As for entities in charge of the execution of the service, the proportion of municipalities where the city government was the only or one of the entities fell from 57.1% in 2008 to 46.2% in 2017. The presence of state companies as responsible for the service changed from 32.4% to 41.6%, of the municipal authorities, from 9.6% to 11.0% and of the private companies from 2.1% to 3.1%.

In relation to the charging of those basic sanitation services, water supply was charged in most of the municipalities where it existed in 2008 (94.0%), changing to 94.6% of the localities with active services in 2017. The charge for sewage disposal was lower, changing from 55.1% in 2008 to 63.9%, in 2017.

As an economic instrument of social policy to guarantee universalization of the access to basic sanitation, the subsidies (discounts) for users are present in 72.6% (3,783) of the municipalities with water supply and in 67.8% (1,387) of those with sewage disposal, where there was charge of a tax or tariff for the respective services. In northern municipalities, for both services, this social policy instrument existed in fewer than 40% of the localities where there was a charge of tax or tariff. The biggest percentages were observed in the Northeast, where there were subsidies to users of the supply services in 79.2% of the municipalities with charge; and in the South, where this proportion was of 79.3% of the municipalities for the sewage disposal service.

The most common criteria for the concession of subsidies were enrollment in social programs (69.6% of the municipalities with subsidies for water supply and 52.8% for sewage disposal), characteristics of the housing unit (63.2% for water and 68.5% for sewage) and earnings of the user or their family (56.8% for water and 63% for sewage). In the country, about 2.8 million household economies (residential units provided with services) received subsidies in the charge of tax or tariff for water supply and 1.5 million in the charge of sewage disposal.

The data comes from the National Survey of Basic Sanitation: Water Supply and Sewage Disposal (PNSB) 2017, which investigates water supply services by a general system in formal entities (with CNPJ) that render this type of service. The publication, tables and presentation area available on the right of the page.

Water supply is close to 100% of coverage

Water supply by a general system was in operation in 5,517 municipalities, and in 33 of them, one or more entities would offer the service, among which only nine had the service already under implementation. In 22 municipalities, the service had been stopped all over 2017, and in another 22 no entity was rendering that type of service. The interruption was mainly due to the occurrence of drought, a phenomenon concentrated in the Northeast, especially in Rio Grande do Norte, where 21 municipalities had at least one entity with such services, and in Paraíba, with 33.

There were, in 2017, 59.8 million active household economies (households with water supply paying bills and/or with consumption) supplied in the country, an increase of 32% against 2008 (45.3 million). That number of household economies corresponds to 86.1% of the total in Brazil. There was also a big difference

between the Major Regions: 47.6% in the North; 73.4% in the Northeast; 90.9% in the Central West; 93.3% in the South and 97% in the Southeast.

Between 2008 and 2017, the percentage of Brazilian municipalities that had water supply by a general system remained virtually the same, with a change from 99.4% to 99.6% (5.548), but with an increase of 3.7 percentage points since 1989 (95.9%). In 88.3% of the municipalities with services in operation (4,873), water is treated in ETAs and/or UTSs. About 75,.% of the treated water distributed in the country goes through conventional treatment, which is lower in less populated municipalities. Lack of treatment is observed in 24.2% of the municipalities with services in operation in the Northeast and in 21.6% in the North Region.

Since 1989, the percentage of municipalities with water supply has been above 90% in all the Major Regions, except for the North, where the service was available in 86.9% of the municipalities in 1989. The data of 2017 show that, in the South and Southeast, water supply services are present in all the municipalities, being roughly at the same level the Central West (99.6%) and the Northeast (99.3%). Water supply reached 98.4% of the municipalities in the North, the Major Region recording major advances, after a result of 86.9% in 1989.

Municipalities with water supply services by system of distribution - PNSB time series - 1989 to 2017

**Major Regions**	**1989**	**2000**	**2008**	**2017**
**Brazil**	**4 245**	**5 391**	**5 531**	**5 548**
North	259	422	442	443
Northeast	1 371	1 722	1 772	1 781
Southeast	1 429	1 666	1 668	1 668
South	834	1 142	1 185	1 191
Central West	352	439	464	465

Source: IBGE, Diretoria de Pesquisas, Coordenação de População e Indicadores Sociais, Pesquisa Nacional de Saneamento Básico 1989/2017.

Among the 5,517 municipalities with watersupply by a general distribution system in operation, the interruption of supply for six or more hours was reported by 2,454 (44.5%), whereas rationing took place in 1 146 of them (20.8%). Both phenomena were most common in the Northeast, where 67.7% of the localities had intermittent supply, and 42.5%, rationing.

Ocurrences of intermittent supply were short, in general: in 1,441 municipalities (58.7% of those with such occurrences), one or more entities reported a durantion of one day, at most; and 871 (35.5%), of two to three days. Short periods of intermittent services can be of minor seriousness as long as housing units have water reservoirs or tanks for that purpose, but can be very trase of housing units without such options. Rationing, however, took longer: in 392 municipalities (34.2% of those where rationing took place) one or more entities reported occurrences not surpassing 10 days, but, in 299 (26.1%), there was rationing for more than 6 months.

Water loss remains at 40% of the abstracted volume

Water supply services encompass the activities of abstraction, treatment and distribution of water to the final consumer. Between the entry to the system and the consumer a roughly 40% of the volume abstracted is lost. In 2017, the total abstracted amounted to 52.4 million m³/day (50.98 m3/day of fresh water and 1.47 m3/day of brackish water); 45 million m³/day treated, 46.1m³/day distributed (43.6 m³/day treated and 2,5 m³/day not treated) and only 26.6 million m³/day consumed.

Almost 70% of the water abstracted in the country is fresh water coming from surface sources, whereas 25.5% comes from deep wells. Only 2.7% is brackish, saline or salt water. In the Northeast, however, abstraction of brackish water amounts to 10.2%. In 2017, the abstracted total amounted to 51 million cubic meterswater and 1.5 million m³/day of brackish water.

40% of the municipalities have no access to sewage disposal services

In nine years, sewage disposal by a general system changed from 55.2% municipalities in 2008 to 60.3% in 2017. That year, there were entities with services in operation in 3,206 of the municipalities, and with services under implementation in 175 (with services under implementation as the only type in 153).

In 2,211 municipalities (39.7%) the service is inexistent. The Northeast and South recorded the highest frequency of municipalities without services in operation: 915 and 704 respectively. In the Southeast, that number falls to 68, representing only 2.9% of that service in the country. In 2,013 municipalities, there are ETEs in operation, and in 1,193 there are no treatment stations.

**Municipalities with sewage disposal by a general system - PNSB time series - 1989 - 2017**				
**Major Regions**	**1989**	**2000**	**2008**	**2017**
**Brazil**	**2 091**	**2 877**	**3 069**	**3 359**
North	25	32	60	73
Northeast	381	767	819	945
Southeast	1 301	1 574	1 586	1 609
South	335	451	472	531
Central West	49	80	132	201
**Source:** IBGE, Diretoria de Pesquisas, Coordenação de População e Indicadores Sociais, Pesquisa Nacional de Saneamento Básico 1989/2017.				

Coverage of sewage disposal services rendered by a general system is lower than that of water supply services, and much more heterogeneous, among the Major Regions. In 2017, whereas in the Southeast more than 90% of the municipalities had that type of services the proportion was only 8.4% in the North in the same year. In spite of that, the Region has almost doubled the service since the start of the series (16.2% in 2017). Also in the Northeast, the increase was similar: the proportion of municipalities with the service more

than doubled, having changed from 26.1% in 1989 to 52.7% in 2017. The best performance was that of the Central West, where municipalities with sewage disposal changed from 12.9% in 1989 to 43.0% in 2017.

In the South, the advance was much more limited, contrasting with other socioeconomic indicators of that Major Region, which, in general, are positive in relation to the other ones in the country. It was observed that in states such as Santa Catarina and Rio Grande do Sul, a common form of sewage disposal is by means of storm drains, sometimes as long as households treat their sewage before by means of individual septic tanks.

There is, in Brazil, a volume of 14.3 million m³ of sewer disposed per day, out of which 11 million m³ are treated, being equivalent to 77.1%. As for the level of treatment (preliminary, primary, secondary and tertiary, in growing order of efficiency), 69.8% of the volume received a type of secondary treatment (oxidation of organic matter by microorganisms), 21.9% of tertiary treatment (removal of pollutants such as nutrients, pathogenic elements, inorganic solids dissolved and in suspension, 5.9% of primary treatment (removal of sedimentable solids in suspension and fluctuating solids), 2.4% of only preliminary treatment (removal of oil, fluctuating dirt and sand).

State companies for sewage disposal services were present in a bigger number of municipalities

As for entities responsible for the execution of water supply services, in 2008, state sanitation companies and municipal authorities were the executing agencies in, respectively, 66.4% and 9.3% of the municipalities. In 2017, these percentages changed from 69.5% and 10.3%. City governments, private companies and associations were the executors in, respectively, 41.6%, 4.5% and 13.1% in 2008; in 2017, they executed the service in 35.2%, 3.6% and 11.6% of the municipalities, respectively.

As for entities responsible for the execution of sewage disposal services, the proportion of municipalities where the city government proportion of municipalities was the only or one of the executors of the service fell from 57.1% in 2008 to 46.2% in 2017. The presence of state companies as executors of the service changed from 32.4% to 41.6% of municipal authorities (Autonomous Water and Sewarage Services - SAAEs), from 9.6% to 11.0% and of private companies from 2.1% to 3.1%.

Private companies are present in 3.6% of the municipalities in the case of distribution of water and in 3.1% in the case of sewage disposal. The proportion observed in water supply is, in fact, lower than the proportion registered in 2008 (4.5%). The decrease was mainly due to a movement of reestatization that took place in the State of Tocantins, where, in 2008, 126 municipalities had the service rendered by a private entity, against only 52 in 2017.

Concession and program contracts are the main delegation instruments

Municipalities can render services directly or delegate them. The main delegation instruments are concession, program contracts or decree laws. Among state sanitation companies, the main instruments delegation of water services are concession (1,636 contracts or 42.7%) and program contracts (1,260 or 32.9%); and for sewage disposal services, program contracts (639 or 47.9%) and concession (539 or 40.4%).

In the case of SAAEs, the predominant instrument is a law or decree, both for water (85.7%) and for sewage (88.4%). In this case, the municipality renders the service indirectly, whereas private companies operate by means of concession for water in 85.0% of the cases (170 municipalities) and for sewage in 94% (94 municipalities).

In private associations, the most common is service-rendering without the existence of any delegation instruments. In general, these associations are very simple and their services are a fruit of the organization of residents in order to make up for the absence of public services.

As for the existence of regulation agencies for sanitation services, it was observed that water supply was regulated in 87.3% of the state companies, 65.0% of the private companies, 36.4% of SAAEs and 5.2% of city governments. Sewage disposal was regulated in 89.4% of the state companies, 73.3%% of the private companies, 4.5% of the SAAEs and 4.3% of city governments.

With reference to the existence of due period and regulation agency, a major part of the delegation instrument is in accordance with the legislation, especially regarding sewage disposal services. On the other hand, the existence of universalization is less common, mainly in municipalities in the Northeast Region.

Both for water supply and for sewage disposal, most of the contracts will be due in 2020 and 2050, being a highlight the decade of 2030s. The average duration of these contracts is 30 years, in the case of water supply, and of 31 years, in the case of sewage disposal.

Total household economies with water supply by a general system advance 32%

There were, in 2017, 59.8 million active household economies (households with water supply paying for a bill and/or with consumption) supplied in the country, an increase of 32% in comparison with 2008 (45.3 million). That number of household economies corresponds to 86.1% of the housing units in the country. Big changes were also observed among the Major Regions: 47.6% in the North; 73.4% in the Northeast; 90.9% in the Central West; 93.3% in the South and 97% in the Southeast,

Each unit, either residential or not, covered by a general system, consumes, on average, 420.1 liters per day. Considering that most active economies is in households (91.4%) and the average of three residents per housing unit, there is a daily consumption of 140 liters per day per resident. The United Nations Organization (UN) recommends consumption of 110 liters/day per resident. All the regions are above that figure.

In relation to sewage disposal, there was an increase of 39.2% of housing units covered. In 2017, active household economies corresponded to 50.8% (50.8% (35.3 million) of the total permanent private housing units in Brazil, versus 43.6% in 2008 (25.4 million).

Charge for water services reaches 94.6% of the municipalities and, for sewage disposal, 63.9%

The percentage of municipalities with services in operation charging for water supply remained stable, with a change from 94.0%, in 2008, to 94.6%, in 2017. In the South, it amounts to roughly 100%; and in the North, with the lowest percentage, there was a slight decrease from 86.4% to 86.%.

Regarding sewage disposal, the national index rose from 55.1% in 2008 to 63.9% in 2017. The biggest expansion came from the North and Northeast. The Central West had the biggest percentage of municipalities, with 88.2%, and the Northeast, the lowest, with 39.9%.

Among the executing agencies of water supply, there were 7,465 registries of entities charging fares in the municipality surveyed, out of which 5,431 have minimum fare.

Water supply subsidies hit 79.2% of the municipalities in the Northeast

Subsidies to users were present in 3,783 municipalities with water supply and in 1,387 of those with sewage disposal, corresponding to 72.6% and 67.8% of the total municipalities where fares or fees were charged for the respective services. Proportionally, in the municipalities of the North, that social policy economic instrument was less frequent: for both services, less than 40% of the localities where there was charging of fares or fees were covered. The Northeast, in turn, was a highlight in terms of water supply services, for which some users received subsidies in 79.2% of the municipalities; and the South, in relation to sewage disposal services, for which that took place in 79.2% of the municipalities. For both services, the most common type of subsidy, which was almost hegemonically distributed, was discount in the fare or fee.

In general, subsidies are granted only to economies served and that fulfill some preestablished criteria. Those should be defined by the holder, the service renderer and the regulating agency, and may vary according to municipalities and executing agencies. They should, however, always be aimed at universalization, in such a way that the beneficiary should have a progressive character and encompass that with a smaller capacity of payment.

BRAZIL. Ministry of Health. Pan-American Health Organization. Health impact assessment of sanitation actions: conceptual framework and methodological strategy. Organização Pan-Americana da Saúde. - Brasília: Ministry of Health, 2004.

The present methodological proposal for evaluation is situated, historically, in a context characterized by important particularities, dictated by the uniqueness of a project with unprecedented scope and diversity in the most recent initiatives in the sanitation sector in Brazil. More than the development of an evaluation of a sanitation program, this project can be seen, fundamentally, as a double opportunity. On the one hand, to evaluate a federal sanitation program that contemplates a wide range of situations provided by the magnitude of the actions. On the other, the perspective of inaugurating a systematic practice of evaluation of sanitation actions and programs in the country, from which will be developed and validated a methodology that, although involving different areas of technical and scientific knowledge can be reproduced at the local level without prejudice to its scientific rigor.

Additionally, a side benefit that should not be neglected is the possibility of building a database on the systems construction of a database on the systems that are the object of the evaluation, which can subsidize the construction of sanitation information systems articulated with epidemiological and economic information, useful for the formulation of general policies for the sector.

Considering also that the interventions in environmental sanitation, financed by the Ministry of Health/FUNASA, were conceived with the purpose of reducing regional inequalities and increasing the quality of life of the benefited populations, having as one of its objectives to raise the level of health of these populations, it is understood to be essential to evaluate the impact that the improvement of the conditions of environmental sanitation may produce in the reduction of morbidity and mortality of the populations.

This procedure, not only may come to represent a landmark in the country's governmental sphere, with regard to the practice of evaluating the effectiveness of its policies and actions, as well as to create opportunities to correct or improve these actions and, above all, to provide clear, transparent answers and transparency to the population about the effectiveness of this public policy.

It is also important to emphasize the magnitude and diversity of the sanitation actions that are the object of this evaluation. It is precisely in this wealth of situations that we find the favorable circumstance to carry out evaluations and to extract information to correct the course of this and future sanitation programs, as well as to accumulate knowledge for the formulation of broader sectoral policies. On the other hand, this diversity increases the challenge of setting up an evaluation methodological proposal that can contemplate the various aspects in an integrated way.

From this perspective, the methodological conception presented in this document was developed. It was built by a multidisciplinary work team that involved technicians, leaders and advisors from the sponsoring institutions and scientific teams in the area of sanitation, epidemiology, anthropology, and health economics. Considering the unprecedented nature of the task, we tried to make clear both the theoretical foundations for the construction of the evaluation and the methodological strategies to be employed in its execution.

Chapter 2 – Objectives

The general objective of this methodological proposal is to evaluate the sanitation actions developed by the Ministry of Health/National Health Foundation (MS/FUNASA) and their impacts on the health of the populations of the municipalities benefited.

The general objective unfolds in the following specific objectives:

- To evaluate the implementation of intersectoral sanitation actions with a focus on the management of services, the sanitary conditions of the localities where the actions will be implemented, and the technological aspects of water supply systems, sanitation improvements in homes, and sewage systems;
- Understand the actions of environmental sanitation in their connections and their unfolding in the way of life and health conditions of the populations involved;
- Assess the impact produced by sanitation interventions on morbidity and mortality by some diseases that have their occurrence related to sanitation;
- Evaluate the cost-benefit and the cost-effectiveness of sanitation actions.

Chapter 5 - Conceptual Framework

Introduction

Institutional and public policy evaluation has become a fundamental and indispensable instrument in the process of formulating and improving the State's actions in the field of social policies. Despite the consensus on the need to promote such evaluations, the conceptual and methodological framework that underlies this activity is still incipient (Belonni et al., 2000). For Rico et al. (1998) the evaluation of social policies and programs has become a challenge for both research centers and centers as well as for governments, due to the need to innovate evaluation concepts and methodologies due to the rejection of "traditional models that fail to grasp, in their entirety, the flows and nexus between decision making, its implementation, execution, results and impacts produced". Due to the scarcity of theoretical literature and evaluative experiences, Belonni et al.(2000) recommend a careful conceptual and methodological discussion when developing evaluation tools. On the other hand, the complexity of contemporary political and social phenomena has demanded an interdisciplinary approach in the evaluation process of public policies.

These evaluations involve the analysis of the processes of formulation, development, actions implemented or facts that have occurred, results and impacts obtained, and also the analysis of the historical and social contexto in which it is conceived. It is not restricted, therefore, to the comparative examination between what is proposed and what has been achieved (Belonni et al., 2000). An evaluation process involves a judgment, concerns the assigning a value to a particular government policy or program (Contandriopoulos, 1994; Hartz, 1998). For Arretche (1998), evaluation is a measure of approval or disapproval of a public policy or program, according to a certain conception of justice.

Figueiredo & Figueiredo (1986) make a distinction between policy evaluation and policy evaluation. The former aims at analyzing and elucidating the criteria that underlie a policy, the reasons that make it preferable to another, and that it should contribute to the welfare of the population. This type of evaluation does not enter into the merits of the appropriation of the benefits of the policy by the population. Moreover, the evaluation examines the political nature of the decision-making process that

led to the adoption of a particular policy, as well as the values and political criteria that guide it, regardless of the institutional engineering and its results (Rico et al., 1998).

On the other hand, policy evaluation seeks to examine the institutional engineering and of programs (Rico et al., 1998). This type of evaluation seeks to elucidate whether the policy is consistent with principles of political and social justice that are minimally accepted and about which there is a minimum consensus, and whether there was an effective appropriation of benefits (Figueiredo & Figueiredo, 1986).

Thus, an evaluation model must be based on a paradigm of society that is articulated with the dynamics of reality production, going beyond simple data collection. Such a paradigm must accompany the changes in social and urban processes and the deeper changes in the conception of Society and development style (Borja, 1997). When referring to the construction of an indicator system model for reality assessment, Pfaff (1975) argues that it should be more broadly defined as a control model, with a set of goals, options, parameters, variables, among others.

Evaluation of Public Policies

Although it is a very old activity, only recently "evaluation" has been gaining new contours, involving multiple applications, especially when it comes to the evaluation of public policies, as a modern societies to obtain the maximum efficiency and effectiveness in the application of public efficiency and effectiveness of public investments.

The area of Economics was a pioneer in the development of methods to indicate the advantages and costs of public programs. However, the approaches employed in this field of knowledge have proven to be insufficient for the evaluation of social programs, especially those in health, sanitation, and education.This finding has stimulated initiatives aimed at developing new methodologies from an interdisciplinary perspective (Contandriopoulos et al., 2000).

In modern societies, public interventions are part of the everyday life of individuals, one of the functions of the State is to formulate and implement policies that improve the living conditions of the population. However, since in developing countries, in general, public resources are quite scarce in relation to the needs, it is inevitable that the State have to make choices about the priority problems to be addressed as well as the most cost-effective technologies(Contandriopoulos et al., 1994). On the other hand, since the expected beneficial effects are often difficult to perceive and measure, especially when such complex objects as health and sanitation are involved, it is necessary to conduct evaluations of these interventions, with a multidisciplinary character, using quantitative and qualitative methodologies.

For several reasons, whether political, technical-scientific, or managerial, evaluating social interventions represents a relevant activity for the decision-action process, as it produces information that managers and funders, to define priorities, to present to society the results of investments made, and to point out modifications and adjustments needed to the interventions already implemented.

Alongside this pragmatic perspective that results from evaluative research, there are other major advantages involved in its realization. Firstly, by involving those responsible for the formulation and implementation of policies and, more broadly, society in general, it makes it possible to broaden the interactions among these various actors and mediates these interactions with qualified technical-scientific information. Another no less important is that the evaluative research constitutes an important source of knowledge about elements of the dynamics of this society itself. It is known that many of the social policies, by having the central purpose of implementing interventions that bring increments in the welfare state of a population, are in fact modifying the population, are in fact modifying the determinants that are at the basis of the initial levels of well-being or health of this population. By producing knowledge about how the intervention changed the previous state of well-being, evaluation is also helping to understand how the determinants act.

In the health care field, it has become a consensus that it is necessary to evaluate the effectiveness of curative or preventive technologies before recommending their adoption by the health system.

Nowadays, it is not acceptable that any new health technology, such as a vaccine or a drug, is introduced for use by the population without going through formal systems of evaluation.

Public health policies and programs are complex interventions that operate multiple technologies, but they cannot be confused with the mere sum of them, and therefore must be subject to specific evaluations. An immunization program, for example, although it is based on one or more vacines (which, in order to be adopted, must have their efficacy proven), it cannot be confused with them. When an immunization program is evaluated, new information will be generated, including the effect of these which includes the effect of these technologies under actual conditions of use, i.e. their effectiveness. A effectiveness, besides being dependent on the efficacy of the technology, also depends on several elements of it,such as adverse effects, as well as others linked to the implementation of the program such as costs and operational difficulties, among others.

A large part of the technologies restricted to the health field are directed to interfere in the course of already installed diseases, and a smaller number to prevent them and thus reduce their occurrence. Distinctly, a series of social or economic public policies have potential effects on health by acting on the the processes that generate the occurrence or determinants of diseases or other health events, thus directly affecting the incidence of these events. Among these, it is worth mentioning the environmental sanitation interventions that, by providing improvements in the hygiene levels of individuals and their context,reduce the contact of the population with a wide variety of vectors, reservoirs, and inanimate vehicles of pathogens and, therefore, reduce the pathogens and, thus, reduce the chances of getting sick from various diseases. Furthermore, these interventions, by providing easily accessible water, as well as more adequate means for the collection and disposal of sewage and solid waste, raise the quality of life and influence the way of life of the populations, which ultimately can also have positive effects on their well-being and health, and health and, indirectly, in the reduction of several diseases related to physical wear.

Complexity of the objects of this study: Health and Sanitation

Despite its routine use and with different meanings, there is no unified concept of health, so its use is always accompanied by a series of inaccuracies that create difficulties of order conceptual, methodological or operational nature. The available definitions are situated in a great diversity, and range from the most comprehensive and operationally intangible (the state of complete physical wellbeing), to those that are conceptually limited, while operationally useful (the absence of disease). Another aspect to highlight is the fact that the health levels of a population are determined by multiple factors, which means to say that the health conditions prevailing in a population at a given moment of time result from a complex interaction of factors of various categories acting on the population. Evaluation, therefore, constitutes an effort to separate the effects of the intervention in foco from a series of other factors and interventions that are continuously acting on populations. This separation is, without a doubt, the main difficulty involved in the evaluation of social programs.

Environmental sanitation is a set of actions aimed at providing increasing levels of environmental health in a given geographic area, for the benefit of the population that inhabits this space. These actions, if adequately implemented, can produce a series of positive effects on the well-being and health of the benefited populations. However, more than this, and as a result of the different effects it provides, adequate environmental sanitation is considered a constituent part of the modern way of life and one of the fundamental rights of citizens of societies.of contemporary societies.

In today's so-called developed societies, improvements in the environmental conditions in which their populations lived evolved in parallel with the intense social and economic development observed since of the end of the 19th century. Therefore, they are intrinsically and inseparably interconnected to this intense process of transformations that has taken place in such societies from that moment until the present day. The so-called developing societies, on the other hand, had their transformation processes quite different from those previously referred to above, having generated as a consequence what is currently one of its main characteristics: the maintenance of significant portions of its populations

without access to goods and services that are considered fundamental rights in the modern way of living. Besides all the negative effects, the fact that portions of the population in this group of societies have gradually gained access to these goods and services, creates new situations in which inequalities that already existed between nations, come to exist within each society. As a result, it becomes less and less tolerable that large parts of society do not have access to these goods and services. As a consequence, the governments of these countries are permanently pressured to implement specific policies and actions that provide adequate sanitation environments for important portions of their populations and, in this way, contribute to reduce the existing inequities.

Brazil has huge deficits in this area, since significant portions of its population do not have access to the benefits of environmental sanitation. This situation is visible both when comparing the regions of the country. It is at the base of the great inter and intra-regional, intra-urban, and urban-rural differentials observed in living and health conditions. Not by chance, sanitation policies have been identified as priorities that could reduce these huge differentials, becoming an important factor for equity.

This theme deepens the question around the spatial inequality verified in the distribution of sanitation services , pointing in the direction of differentiated access to the networks by the population, since the existence of the network does not always mean a broad service to the population, given its small extension in relation to the number of households served. In this context, the universalization of sanitation services is the major goal to be achieved in the country. The sanitation program, the object of this evaluation proposal, despite its magnitude and diversity, involving municipalities from all regions of the country does not fill the enormous deficits accumulated in the sector, especially with regard to sanitary sewage.

Health and Sanitation Assessment

The fact that it is possible to implement extensive sanitation programs in relatively short periods of time makes their effects differentiated with respect to the set of changes that are taking place in a given Society, which makes it possible that these interventions can somehow have their effects identified and eventually, measured.

Evaluative research should broaden the existing evidence base on the issue and provide plausible explanations for the results found by examining implementation procedures and investigating contextual mediating factors. As there is no known precedent in the literature for evaluating a sanitation program of this scale and of a sanitation program of this scope implemented in a relatively short period of time, and that, in addition, proposes to address so many aspects, it is evident that the effort to overcome the difficulties of its realization will have to be faced. by the inclusion of several axes that will use different methodological resources, but it is expected that the final results will provide an integrated set of useful knowledge.

In this way, this evaluation should not be considered as a single study, but as an organized set of studies, and the complexity of the of studies, and the complexity of its object requires the analysis of several axes and the involvement of different disciplines and strategies, in order to obtain consistent and reliable results.

Axes and Dimensions of the Assessment

The present evaluation proposal is based on axes that use different methodological resources, in order to contemplate the various interfaces of the health and sanitation relationship. In a discussion process conducted by PAHO and the Ministry of Health, the following axes were defined: **Sanitation, Anthropology, Epidemiology** and **Health Economics.**

This set of axes includes some six dimensions of analysis that aim at evaluating particular aspects of the impact of sanitation actions on health, translated into specific issues of infrastructure, organization, costs and benefits of sanitation actions; perception of the population involved; and epidemiological impact. In general, these dimensions portray the scenario in which the sanitation actions will be implemented, seeking to capture and assess the various relationships between man and the environment.

The sanitation axis have 3 dimensions: management of services, sanitary conditions and technological aspects.

Sanitation Services Management Dimension

According to Moraes (1997), the management system of public sanitation services is formed by the set of institutional agents, governmental and private entities, which have the objective of executing the adopted sanitation policy with the sanitation plan as its main instrument. For the author, the management of the sanitation services must be supported by a sanitation policy, in which its general guidelines, its management model, the legal and institutional organization and the management system that gathers the instruments for planning, execution, operation and evaluation of the sanitation works and services, according to the principles of a public sanitation policy. Moraes (1997) also considers that governmental actions are reflected in laws, decrees, rules and regulations in force.

Dimension of Sanitary Conditions

The term "sanitary condition" expresses the level of environmental salubrity, if related to the existence of hygienic conditions of the dwellings and of the public space, involving from household sanitary plumbing installations to public sanitation systems. Sanitation systems involve various individual and collective solutions for water supply, sewage and solid waste disposal, and drainage of rainwater. These systems must have sufficient quality and quantity to promote public health and control environmental pollution.

Dimension of Technological Aspects

This evaluation dimension includes aspects related to the technologies used in the sanitation projects, as well as the process of implementation of the works and services and the subsequent operation of the projects and facilities executed. It is considered that the technologies must be appropriate to each reality from the sociocultural and environmental point of view, in order to obtain effectiveness in the use and operation of the works and services implemented and efficiency in the implementation process with respect to costs and the physical and financial schedule. In this aspect, the methodological support and the integrated work with the **Anthropology Axis**, **responsible for the sociocultural dimension**. Although, in the last decades, the technical the technical-scientific community of sanitary engineering has awakened interest and made an effort to adopt more appropriate technologies to each local reality, there is still a long way to go to ensure that, effectively, the projects are conceived and implemented according to this principle.

The **Epidemiological Axis** has **epidemiological aspects** as **dimension**, which has as objective to evaluate the impact produced by sanitation interventions on morbity and mortality from some diseases that have their occurrence related to sanitation.

The health economy axis has as dimension health economic evaluation.

Health Economic Dimension

This dimension seeks to evaluate the efficiency of the interventions in sanitation with regard to the scope and results of the implemented actions, especially in terms of improvements in epidemiological and sanitary indicators. It also makes it possible to verify whether the benefits generated by the implemented measures are distributed equitably among the population.

The performance of economic evaluations, in turn, requires the selection of objective instruments that and guide social investment decisions. In this sense, the choice of available economic evaluation requires a clear knowledge of the phenomenon in question, and the most pertinent methodological design will depend largely on what the investigation wishes to identify.

Cause and effect explanatory model

As shown above, there is a high degree of complexity in the health-environment relationship, which can be assessed by focusing on different dimensions. This relationship can be seen as a cause and effect relationship in which certain conditions, associated with the lack of sanitation, end up generating negative effects on health.

To explain this causal relationship, models are often used that represent an effort to approximate reality. To portray the complex relationship between health and sanitation, we chose to adopt the explanatory model proposed by the World Health Organization - WHO (Corvalan et al,1996), structured as a chain of cause and effect, known as DPEEEA - Driving Forces, Pressures, States, Exposures, Effects and Actions.

The DPEEEA model

The World Health Organization (WHO), concerned about the impact of compromised environmental health on human health, has promoted studies to better understand the relationship between environment and health in order to support the definition of policies and strategies for these sectors. Within this effort, the project HEALDLAMP (Health and Environment Analysis for Decision-making) project, which aims to "improve information support for environmental health policies and the provision of information about the impacts of environmental health at various levels to decision makers, health professionals, and the public" (Briggs et al, 1996).

Based on an adaptation of the Pressure - State - Response framework, used in the construction of the Organization for Economic Cooperation and Development – OECD (based on work developed by the developed by the government of Canada), the WHO proposed the conceptual framework for environmental health indicators. The DPEEEA (Driving Forces, Pressures, States, Exposures, Effects and Actions) model seeks to explain how various driving forces generate pressures that affect the state of the environment that expose the population to risks and affect human health (OECD, 1993).

The cause and effect matrix proposed by the WHO is represented by a chain entitled Development - Environment - Health, which reveals the understanding that health is the result of the interaction between development and environment. Thus, for example, the driving forces of development, represented by urbanization and industrialization, generate pressures on the environment that deteriorate its state and expose the population to risks, which can generate negative effects on human health.

The Ministry of Health, responsible for implementing the Unified Health System (SUS), has as one of its responsibilities to protect the population from damage to health. In this perspective, it has been financing sanitation actions on a large scale in order to reduce risks resulting from the deterioration of the urban environment. The FPEEEA model fits the main objective of this project, which is to measure the effects of these interventions on the health of the benefited populations, which is why it was adopted in this evaluation. It is noteworthy that this methodology foresees the definition of indicators at various points in the chain.

As already explained, there is a high degree of complexity in the relationship between health and environment. Besides that, every model brings in itself limitations, because it is an attempt to approximate and explain reality. That is, whatever the model adopted, it is not possible to grasp reality in its entirety, because it is the product of a historical, economic, and social process. The chain of cause and effect adopted partly fulfills this role, as it has proven useful in trying to explain the relationship between health and environment.

In view of the complexity of the phenomena that involve this evaluation and, furthermore, considering that not all phenomena can be measured through indicators, we intend to privilege in our model the sanitation actions and some effects over others. In principle, due to the main objective of this evaluation, we privileged the effect on human health, although we also considered the effects on the environment, social exclusion, and citizenship.

Conclusion Chapter

This publication is the result of the conception stage of the health impact assessment model of sanitation actions.This proposal is the fruit of the construction process of the evaluation in question, in which the insertion of the different axes, Sanitation, Anthropological, Epidemiological, and Economic, took place gradually, as a result of a rich and fruitful discussion environment. The text also included the conclusions of several meetings of the Task Group and Workshops held from 2002 to 2004, which resulted in the enrichment of its content.

It is worth highlighting, in this process, the role of the institutions involved, which, due to their history of work in the health-sanitation relationship, created a favorable environment for the development of the work. These institutions, notably PAHO, FUNASA, and SVS, were clear and competent to conduct the methodological development in the transition period between two federal administrations, doing so in a transparent and well-founded manner, resulting in a proposal with credibility in the sphere of public administration.

Another important aspect was the selection process of the research institutions responsible for development of the work. A public bidding process resulted in the choice of university institutions with experience in the area, which has been of great importance for the scientific credibility of the proposal. It is worth noting that, in general, the various forums where the discussion of this proposal was held, such as the ABRASCO Congress, have indicated that the model conceived for the evaluation is adequate, with emphasis on the idea of using the FPEEEA model as the common thread between the causes and effects due to inadequate environmental sanitation on health (expanded morbidity and mortality). Although not the main focus of this proposal, it became evident the possibility of expanding the study to two other suggested effects, namely: (a) environmental effects (compromised ecosystems) and (b) socio-political effects (social exclusion and reduced levels of citizenship).

Although the methodological conception for the evaluation was considered adequate, with the use of indicators as evaluation elements, it should be noted that the importance of constant feedback on the evaluation project was evidenced. This adjustment should be made throughout the development of the work, with the case studies as the fundamental basis. In addition, it is important to emphasize that the indicators, in general, will deserve a sensitivity analysis. According to the dialectical strategy adopted in the development of the evaluation project, the first results of the research establish guidelines for the confrontation between the present evaluation model and reality, allowing the correction of important aspects for the reality, allowing important aspects to be corrected for the continuity of the research execution and the analysis and interpretation of the results.

Integrated evaluation is a crucial aspect to consider. Although the evaluation proposal, for methodological issues, has been conceived in different axes, its integrated evaluation should cover all dimensions involved. The evaluation dimensions contemplated in the present work are: management, health and technology, sociocultural, epidemiological, and economic. Therefore, it is fundamental that the global evaluation manages to integrate these partial evaluations, trying to approach the political-institutional dimension. As an example, the measure of effectiveness of sanitation actions can be the result of the impact on health, which is verified by epidemiological methods. In the same way, the efficiency of these actions may be evaluated by the economic dimension. The integrated evaluation, by including all the dimensions, will make it possible to indicate the sustainability of sanitation actions implemented by government programs.

Although this methodological proposal was developed based on a scenario of sanitation actions generated by a specific government program, the goal of the work is to present an evaluation

methodology that can be incorporated by public agents to monitor sanitation actions in general. To this end, it is essential to clearly identify indicators to be incorporated into the regular information surveys, such as the PNSB produced by IBGE, which will ensure a continuous follow-up of the sanitation reality by means of secondary data.

This publication is, in a way, an important milestone in the methodology development stage. The idea is that the conditions are now in place for the evaluation to be carried out and its results analyzed. However, the production of results and their analysis should not be seen as activities limited only by what is pointed out in this methodological proposal. This is because, as elaborate as the process of methodological, it has always been clear that there would be a need for course correction. As possible to predict, the partial evaluations will be developed over a relatively long period of time, which indicates the possibility of evolution in the understanding and practice of a complex evaluation process such as the one presented in this proposal.

Something remains to be said about the validation stage of this methodological proposal. The researchers involved are clear that the same care taken in presenting the methodological proposal should be taken in the validation stage.This means taking advantage of different ways to discuss the results found, such as scientific events and research meetings. One should also aim to publish the results in an integrated manner, allowing for comprehensive criticism of the entire evaluation process.

Supplementary Material S2: Tables S4-S9 complete with calculations of the scores

Indicator	Parameter	Water quality analysis	Participant statements collected from the school community.<br><br>Where: Nº (%)	Score (0 to 1)
**Physical parameters of water quality**	Colour	Samples were 0µH, below the Maximum Allowed Value of 15µH (Brazilian Water Potability Standard: Portaria MS nº2914/2011)	-	1
	Turbidity	Samples were 0.12UT to 0.41UT, below the Maximum Allowed Value, 5.0UT (Brazilian Water Potability Norm Portaria MS nº888/2021)	-	1
	Temperature	Temperature was at a value accepted by the population and found in Brazilian aquatic environments (ie 20 - 30ºC)	-	1
**Physical parameters for perception of water quality (n=25)**	Taste (adequate water is tasteless)	-	11(44%) the water was tasteless.	0.44
	Colour (suitable water is colourless)	-	15(60%) the water was transparent.	0.60
	Odour (adequate water is odourless)	-	19 (76%) the water had no smell	0.76
			**Average physical parameters**	(1+1+1+0.44+0.60+0.76)/6 =0.8
**Chemical parameters for water quality**	pH	Results ranged from 7.4 to 7.7 within the range recommended in the Potability Standard (from 6.0 to 9.0). Portaria |MS No. 2914/2011		1
	Free residual chlorine	Results ranged from 0.06 to 0.08 mg/l indicating residual chlorine below the Potability Standard (0.2 to 2.0 mg/L)		0
			**Average chemical parameters**	(1+0)/2=0.5
**Microbiological Parameters**	Total coliforms	Total coliforms were present: these should be absent in 100 ml of water according to The Brazilian Water Potability Standard.		0
	Escherichia coli	E. coli was absent		1
			**Average microbiological parameters**	(0+1)/2=0.5
**Water analysis frequency: The Ordinance on Water Potability, Ministry of Health, Brazil requires annual sampling (n=6)**		-	1(17%) indicated once a year	0.17
**Food safety (as related to water quality in food preparation)**		The kitchen water sample was contaminated with total coliform, thus not suitable for food preparation	-	0
			**Average**	(0.8+0.5+0.5+0.17+0)<br><br>=1.97/5=0.39

Table S4. Results of the survey carried out for the Sanitary Dimension and its indicators. Weighting was 3; where n=total number of respondents, TU=Turbidity Units.

Indicator	Participant statements collected from the school community. Where: No (%)	Observations during the site visit	Score (0 to 1)
**Frequency of extreme events (drought) (n=6)**	1 (17%) of respondents said that there has been no drought	Annual rainfall index of 641.7 mm with droughts lasting 6 to 8 months. The site is classified as a hot semi-arid climate with a rainfall variation of 250-750mm per year (Brazil, 2020). As the maximum rainfall in Brazil is 1800 mm per year, 641,7mm is equivalent to 35.65%	(0.17*1+0.356)/2=0.263
**Frequency of water supply (4 teachers and 2 GSAs only, n=6)**	3 (50%) of respondents said that water arrives every day. This question was only asked of 4 teachers and 2 SGAs.	Water supply was not regular. Water in the well was brackish; the school was supplied with a water truck every 15 to 20 days.	(0.5+0)*2/2= 0.25
**Quantity of water**	9 (36%) there is abundant water	-	0,36
**Types of water use (n=25)**<br><br>**(n=2)**	12 (48%) water was used for drinking, washing hands, brushing teeth and flushing toilets, it is also used in the kitchen.<br><br>2 (100%) toilet cleaning occurs daily using water and cleaning materials (GSA).	-	(0.48+1)/2=0.74
**Impact of drought on food (n=25)**	7 (28%) the drought does not, or rarely, impacts food	-	0.28
**Impact of drought on cleaning (n=2)**	2 (100%) drought does not harm the cleanliness of the school (GSA)		1
		**Average**	(0.17+0.5+0.36+0.74+0.28+1)/6 = 0.48

Table S5. Results of the survey carried out for the Environmental Dimension and its indicators. Weighting was 2; where n=total number of respondents.

Indicator	Participant statements collected from the School Community where: Nº (%)	Observations during the site visit	Score (0 to 1)
**Water source type (n=25)**	6(24%): water was supplied from a well, considered a safe source according to WHO.<br>5(20%): from a pipe from the street,<br>4(16%): delivered by water tanker.	The well water was brackish and supplies the kitchen and lavatories. There was no frequent water supply.<br>No water was supplied from the street pipe.<br>The tanker supplied fresh water, distributed via the school's drinking fountain.	(0.60+0)/2=0.30
**Water storage type (n=25)**	7(28%): cisterns and water tanks.<br>13(52%): water tanks,<br>1(4%) cistern.	There was a cistern on the ground and a raised water tank.	(0.84+1)/2=0.92
**Maintenance of the water supply system (teachers only; n=4)**	4(100%): the water tank had a lid.	The supported cistern had a lid, but the water tank was uncovered.	(1+0)/2=0.5*1
**Frequency of water tank cleaning (n=6)**	4(66%): water tank hygiene was carried out at intervals of between 1 and 6 months	-	0.66
**Maintenance of equipment used for water treatment (n=6)**	3(50%): the filter candle was changed at intervals of between 6 months to 1 year.	-	0.50
**Type of material used for storage (verified by colour)**<br>**n=25**	11(44%): blue (plastic),<br>5 (20%): white box,<br>2 (8%): cement,<br>1(4%): grey.	The two boxes were made of concrete, with the cistern painted white	(0.76+0.25)/2 *2=0.505
**Water distribution points (n=25)**	3(12%): water was distributed via a drinking fountain, hand washing sink, the shower, toilet, kitchen sink and tank.	The places where water was delivered were verified, ie the drinking fountain, sinks, shower, toilet, kitchen sink and tank. However, there was no filter on the kitchen tap.	(0.12+1)=0.56
**Water consumption points (n=25)**	14(56%): drinking fountain,<br>6(24%): kitchen tap with filter	-	0.80*3
**Treatment, GSA only (n=2)**	2(100%): washing was performed whenever the candle was dirty or every month		1
		**Average**	(0.30+0.92+0.50+0.66+0.50+0.505+0.56 +0.80+1)/9= 5.745/9=0.64

Table S6. Results of the survey carried out for the Technological Dimension and its indicators. Weighting was 3; where n=total number of respondents.

Indicator		Information collected from the School Community: Nº (%) of respondents not experienced symptoms, infections, disease or condition	Health Data (for the municipality)	Score (0 to 1)
**Symptom**<br>**N=25**	Diarrhoea (stomach ache)	6 (24%)	-	0.24
	Bloody diarrhoea	13 (60%)	-	0.60
	Yellowish skin and/or eyes	18 (72%)		0.72
	Red eyes	12 (48%)		0.48
	Fever with chills	10 (40%)		0.40
	Joint pain	8 (32%)		0.32
	Headaches	2 (8%)		0.08
	Abdominal pain	7 (28%)		0.28
	Intestinal pain	11 (52%)		0.52
	Lack of appetite	9 (36%)		0.36
	Nausea and/or vomiting	11 (44%)		0.44
	Toothache	5 (20%)		0.20
			**Average symptoms**	0.39
**Disease diagnosis**<br>**N=25**	Diarrhoea	6 (24%)	(a) 8(2019); 6(2020) and 1(2021) (b) 2(2020)	(0.24+17/35360*1)/2= 0.12
	Verminosis in general	16 (64%)		0.64
	Amoebiasis	17 (68%)		0.68
	Typhoid or paratyphoid fever	16 (64%)		0.64
	Giardiasis or cryptosporidiosis	17 (68%)	a)1(2019)	(0.68+1/35360)/2=0.34
	Cholera	16 (64%)		0.64
	Kidney disease	19 (76%)		0.76
	Hepatitis, infectious	16 (68%)	c)1(2021)	(0.68+1/35360)/2=0.34
	Gastroenteritis	17 (68%)	(c) 7(2019), 10(2020) and 2(2021)*2.	(0.68+19/35360)/2=0.34
	Leptospirosis	19 (76%)		(0.76+1/35360)/2=0.38
	Hypertension	10 (40%)		0.4
	Dental caries	9 (36%)		0.36
	Gingivitis	11 (44%)		0.44
				0.47
			**Epidemiological Dimension**	(0.39 + 0.47)/2 = 0.43

Table S7. Results of the survey carried out for the Epidemiological Dimension and its indicators. Weighting was 3; where n=total number of respondents.

Indicator		Participant statements collected from the school community. Where: No (%)	Score (0 to 1)
**School dropout (n=4)**		4 (100%) no school dropout due to drought	0*[1]
**Absenteeism from work and school**	**(n=6)**	2 (33%) no work missed due to drought.	(0.33 +0.57)/2 = 0.45
	**(n=19)**	12 (57%) no student absenteeism	
**Depression (n=25)**		11 (44%) no cases of depression	0.44
**Children's learning (n=25)**		5 (22%) no change in learning ability	0.22
**Children's concentration (n=4)**		1 (25%) students were not affected during drought	0.25
**Behavioural changes (n=25)**		10 (40%) no behavioural changes during drought	0.40
		**Average**	0.29

Table S8. Results of the survey carried out for the mental well-being Dimension and its indicators. Weighting was 3; where n=total number of respondents.

Indicator	Participant statements collected from the school community. Where: No (%)	Score (0 to 1)
**Daily amount of water intake (n=25)**	6(24%) 2 litres of water per day consumed as recommended by WHO	0,24
**Hygiene habits (brushing teeth, washing fruit and vegetables, eggs, washing hands, wearing gloves and wearing closed shoes when cleaning at school**	7(38%) of students brush their teeth at school (n=19)<br>12(57%) wash fruit, vegetables and greens (n=19).<br>2(100%) wash eggs (n=2).<br>2(100%) GSA wash their hands frequently, wear gloves, wear closed shoes when cleaning and wear a cap to cover their hair when working in the kitchen (n=2)	(0.38+0.57+1+1+1+1+1)/7=0.85
**Use of personal protective equipment (use of gloves and wearing closed shoes)**	2(100%) use gloves when cleaning and wear closed shoes during cleaning (n=2)	1
**Rational use of water awareness (n=25)**<br>**(n=19)**	22 (88%) ration their water use/ turn off taps during and after use.<br>13 (69%) students use water when brushing their teeth or rinsing afterwards.	(0.88+0.69)/2 = 1.57/2 = 0.785
**Raising awareness of the importance of water (n=2)**	2 (100%) GSA showed the importance of water treatment as only clean or filtered water is good for drinking, cooking and brushing teeth.	1*[1]
**Awareness-raising of water (importance of water, know that only clean is to be used, educational events, talking about water in the classroom, knowledge about waterborne disease)**	2(100%) demonstrated care with water, promptly solving leaks and 2(100%) know that only clean water is to be used in cooking, drinking and brushing teeth (n=2)<br>7(28%) said that there are educational events in the school (n=25)<br>19(83%) said that teachers talk about water in class (n=25)<br>16(76%) have heard about water borne diseases due to talks in school, on the radio or television (n=25)	(1+1+0.28+0.83+0.76)/5=0.88
	**Average**	0.83

Table S9. Results of the survey carried out for the Socio-cultural Dimension and its indicators. Weighting was 1; where n=total number of respondents.
